# Lysosomal processing of sulfatide analogs alters target NKT cell specificity and immune responses in cancer

**DOI:** 10.1172/JCI165281

**Published:** 2023-12-21

**Authors:** Kumiko Nishio, Lise Pasquet, Kaddy Camara, Julia DiSapio, Kevin S. Hsu, Shingo Kato, Anja Bloom, Stewart K. Richardson, Joshua A. Welsh, Tianbo Jiang, Jennifer C. Jones, Susanna Cardell, Hiroshi Watarai, Masaki Terabe, Purevdorj B. Olkhanud, Amy R. Howell, Jay A. Berzofsky

**Affiliations:** 1 Vaccine Branch, Center for Cancer Research, National Cancer Institute (NCI), NIH, Bethesda, Maryland, USA.; 2 Department of Chemistry, University of Connecticut, Storrs, Connecticut, USA.; 3 Department of Microbiology and Immunology, Institute of Biomedicine, University of Gothenburg, Gothenburg, Sweden.; 4 Department of Immunology and Stem Cell Biology, Faculty of Medicine, Institute of Medical, Pharmaceutical and Health Sciences, Kanazawa University, Kanazawa, Ishikawa, Japan.; 5 Neuro-Oncology Branch, Center for Cancer Research, NCI, NIH, Bethesda, Maryland, USA.

**Keywords:** Cell Biology, Immunology, Antigen processing, Cellular immune response, Tumor suppressors

## Abstract

In a structure-function study of sulfatides that typically stimulate type II NKT cells, we made an unexpected discovery. We compared analogs with sphingosine or phytosphingosine chains and 24-carbon acyl chains with 0-1-2 double bonds (C or pC24:0, 24:1, or 24:2). C24:1 and C24:2 sulfatide presented by the CD1d monomer on plastic stimulated type II, not type I, NKT cell hybridomas, as expected. Unexpectedly, when presented by bone marrow–derived DCs (BMDCs), C24:2 reversed specificity to stimulate type I, not type II, NKT cell hybridomas, mimicking the corresponding β-galactosylceramide (βGalCer) without sulfate. C24:2 induced IFN-γ–dependent immunoprotection against CT26 colon cancer lung metastases, skewed the cytokine profile, and activated conventional DC subset 1 cells (cDC1s). This was abrogated by blocking lysosomal processing with bafilomycin A1, or by sulfite blocking of arylsulfatase or deletion of this enyzme that cleaves off sulfate. Thus, C24:2 was unexpectedly processed in BMDCs from a type II to a type I NKT cell–stimulating ligand, promoting tumor immunity. We believe this is the first discovery showing that antigen processing of glycosylceramides alters the specificity for the target cell, reversing the glycolipid’s function from stimulating type II NKT cells to stimulating type I NKT cells, thereby introducing protective functional activity in cancer. We also believe our study uncovers a new role for antigen processing that does not involve MHC loading but rather alteration of which type of cell is responding.

## Abstract 2

<p>Unexpected lysosomal glycolipid processing reverses target NKT cell specificity, thereby altering anti-cancer function.</p>

## Introduction

The recent clinical success of checkpoint inhibitors, which manipulate T cell function and extend patient survival in many cases, provides strong motivation to treat cancer by modulating immunity. However, a certain large proportion of patients across multiple cancers still cannot benefit from the existing immunotherapies (1, 2). Therefore, understanding the interactions between tumor development and the immune system and developing other novel immunotherapeutic approaches are urgently needed.

NKT cells are a unique T cell subset that is developmentally and functionally distinct from conventional T cells. NKT cells develop in the thymus and express T cell receptors (TCRs), which recognize lipid antigens presented by the MHC-like molecule CD1d (3–7). NKT cells then follow a subsequent development pathway and are detected in the periphery in a partially activated state, harboring preformed mRNA transcripts coding for several cytokines and allowing very rapid secretion of large amounts of cytokines upon stimulation. Thus, NKT cells serve as components of innate immunity and adaptive immunity and can potentially drive subsequent responses of other immune cells (8, 9).

**Figure 1:**
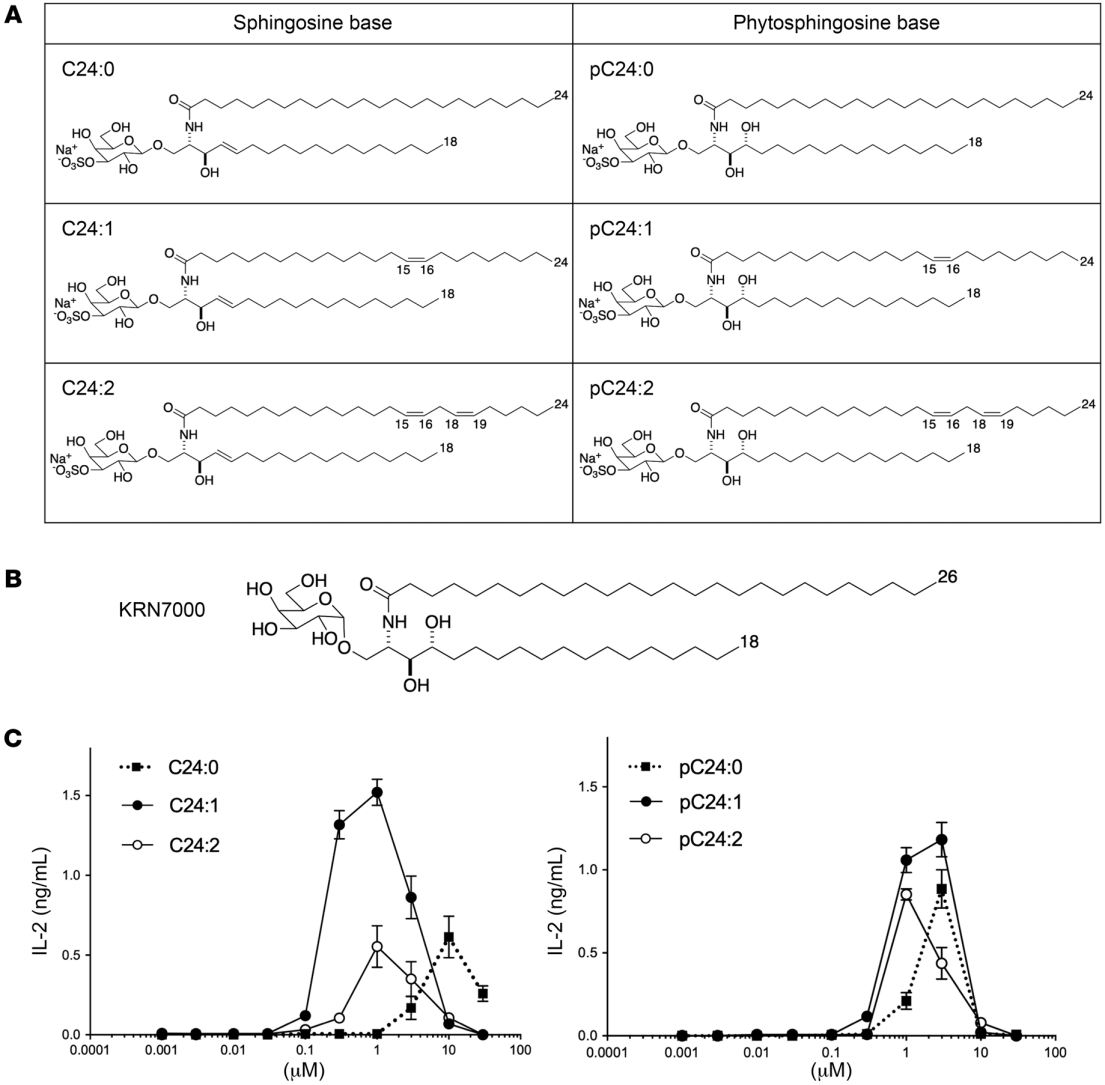
Sulfatide analogs with a sphingosine or phytosphingosine base stimulate type II NKT cells. (**A**) Structures of sulfatide analogs and (**B**) KRN7000 used in this study. Analogs C24:2, pC24:0, pC24:1, and pC24:2 are, to our knowledge, new lipids not previously synthesized or studied as NKT cell agonists. The syntheses of these new compounds (Howell laboratory) are described in detail in the Supplemental Methods. (**C**) The type II NKT cell hybridoma clone XV19 was stimulated for 24 hours with 0.5 μg plate-bound CD1d monomers loaded with graded concentrations of sulfatide analogs with a sphingosine base (left) or a phytosphingosine base (right). IL-2 concentrations in the culture supernatant were measured by ELISA. Data represent 2 experiments (mean ± SD of triplicate cultures).

There are 2 main subsets of NKT cells: type I and type II NKT cells. Type I NKT cells are characterized primarily on the basis of their invariant TCRα expression (Vα24Jα18 in humans and Vα14Jα18 in mice), which is paired with a limited set of TCRβ chains, and their reactivity to the glycolipid α-galactosylceramide (αGalCer) (which we will use to refer to the specific structure of KRN7000; see Figure 1B). On the other hand, type II NKT cells express a different and more diverse TCR repertoire than do type I NKT cells (4, 7). The most widely studied antigen for type II NKT cells is sulfatide. Sulfatide-reactive type II NKT cells are reported to have immunosuppressive functions in experimental autoimmune encephalomyelitis (10), autoimmune hepatitis (11), type 1 diabetes (12), and allergic airway inflammation (13, 14). In tumor settings, we previously reported that sulfatide-reactive type II NKT cells suppress tumor immunosurveillance in experiments showing that the injection of sulfatide increased the development of lung metastasis and inhibited the protective effect of type I NKT cells (15). These suppressive activities of type II NKT cells were mainly reported to be induced by stimulating them with native sulfatide, a mixture of different sulfatide isoforms.

Although many studies have been published on structure-function relationships among αGalCer analogs that stimulate type I NKT cells, including variants that elicit distinct cytokine profiles (see Discussion) (16–24), the functional activities of each component of native sulfatide or synthetic sulfatide analogs have not yet been well described. This study aimed to investigate the functional activities of synthetic sulfatide analogs, especially against tumor immunity, and to gain better knowledge about the structure-function relationship of sulfatides to develop novel strategies for antitumor immunotherapies. The analogs were produced by modifications of the number of double bonds in the acyl chain and the type of sphingoid base in the ceramide structure. Of importance is the discovery that the C24:2 analog with 2 double bonds and a sphingosine base substantially reduced the development of lung metastases. In exploring the mechanisms of this structural effect on function, we discovered that a major factor is how these molecules are processed by DCs. We found that C24:2 stimulated type I NKT cells when processed and presented by DCs, whereas it stimulated type II NKT cells when presented by CD1d monomer on plastic. Although some processing of glycolipid NKT agonists has been described (21, 25), such processing of glycosylceramides to alter their specificity for different target cells and thereby alter their function has not, to our knowledge, been observed previously. We believe that understanding the mechanisms that underlie the relationships between sulfatide endosomal or lysosomal processing and NKT cell functions will lead to promising new strategies for cancer immunotherapies.

## Results

### Sulfatide analogs with either a sphingosine base or a phytosphingosine base stimulate type II NKT cells.

In this study, we evaluated 6 different sulfatide analogs, 4 of which were newly synthesized (C24:2, pC24:0, pC24:1, and pC24:2) (Figure 1A). These analogs are classified into 2 groups on the basis of their sphingoid base, C18-sphingosine or C18-phytosphingosine (26), the latter being the sphingoid base of KRN7000, the prototypical agonist of type I NKT cells (Figure 1B). Each sulfatide contains an acyl chain with 0, 1, or 2 double bonds. The sulfatides are designated C24:0, C24:1, and C24:2 for the sphingosine series and pC24:0, pC24:1, and pC24:2 for the phytosphingosine series (Figure 1A). The sulfatide analog C24:1 is the major component of the native sulfatide mixture in the myelin of the nervous system. C24:1 was shown in previous reports to be one of the immunodominant components of native sulfatide to stimulate type II NKT cells (27–29). First, we investigated the immunoreactivity of each sulfatide analog utilizing the sulfatide-reactive, CD1d-restricted NKT cell hybridoma clone XV19, which was derived from type II NKT cells (30). We stimulated XV19 with the plate-bound mCD1d monomer loaded with each sulfatide analog and measured IL-2 levels in the culture media as an activation marker. Titration curves of each analog showed that, among the sphingosine base group, C24:1 had the highest and C24:0 had the lowest stimulation of XV19 (Figure 1C, left), which is consistent with previous reports (27). In the phytosphingosine base group, the corresponding analogs had a similar rank order of reactivity, with pC24:0 being the least potent and pC24:1 the most, but their reactivity was still weaker than that of C24:1 in both magnitude of response and potency on a molar basis (Figure 1C, right). These results suggest that all the sulfatide analogs tested stimulated type II NKT cells and that C24:1 induced the highest immunoreactivity among the 6 analogs used in this assay.

### C24:1 and C24:2 stimulate type II NKT cells specifically in a CD1d-dependent manner.

**Figure 2:**
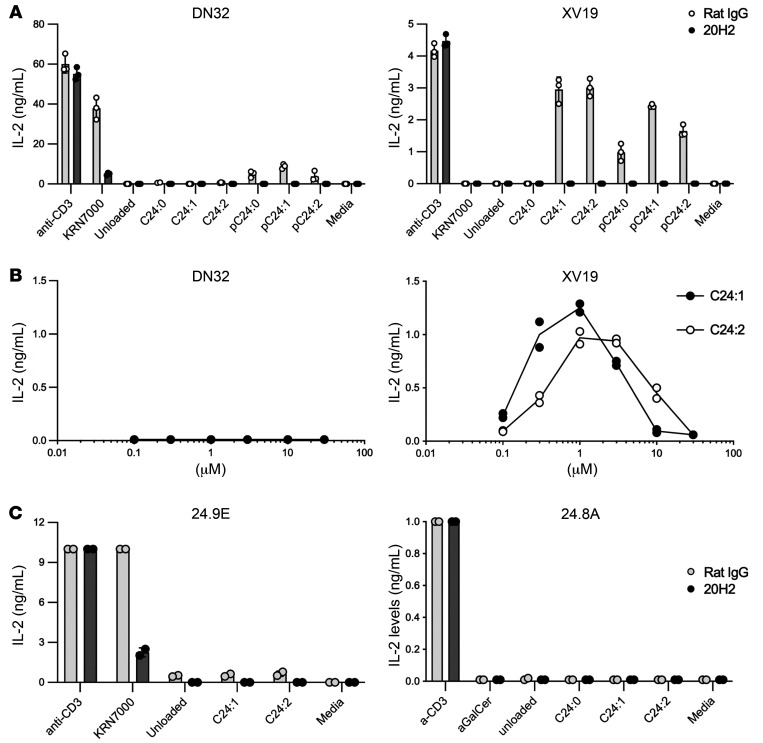
Sulfatide analogs with a sphingosine base stimulate the type II NKT hybridoma clone in a CD1d-dependent manner. Specificities of sulfatide analogs against type I or type II NKT cells were evaluated by ELISA to measure IL-2 concentrations in the culture supernatants of hybridoma clones stimulated with each sulfatide analog. (**A**) The type I NKT hybridoma clone DN32 (left) and the type II NKT hybridoma clone XV19 (right) were stimulated for 24 hours with 0.5 μg plate-bound mCD1d monomers that were either unloaded or loaded with each sulfatide analog (0.5 μM) or KRN7000 (8.73 μM), or with 0.5 μg plate-bound anti-CD3 antibody in the presence of 10 μg/mL rat IgG or anti-CD1d antibody (20H2). (**B**) The type I NKT hybridoma clone DN32 (left) and the type II NKT hybridoma clone XV19 (right) were stimulated for 24 hours with 0.5 μg plate-bound mCD1d monomers loaded with graded concentrations of C24:1 or C24:2. (**C**) The type I NKT hybridoma clones 24.9E (left) and 24.8A (right) were stimulated for 24 hours with 0.5 μg plate-bound mCD1d monomers unloaded or loaded with C24:1, C24:2 (each 1 μM), or KRN7000 (8.73 μM), or with 0.5 μg plate-bound anti-CD3 antibody in the presence of 10 μg/mL rat IgG or anti-CD1d antibody (20H2). Data represent at least 2 experiments and the mean ± SD of triplicate (**A**) or duplicate (**B** and **C**) cultures.

Next, to better characterize each sulfatide analog, we stimulated both type I and type II NKT cell hybridoma clones (DN32 and XV19, respectively) with each sulfatide analog at the same concentration (0.5 μM), presented by the plate-bound CD1d monomer (Figure 2A). Notably, the maximum level of IL-2 secreted from DN32 and XV19 in response to anti-CD3 was substantially different. Likewise, the absolute magnitude of the response to lipids varied between the hybridoma clones, so the magnitudes must be compared only in relation to the anti-CD3 control for that hybridoma, and not between hybridomas. All 3 analogs in the phytosphingosine base group activated both type I and type II NKT cell hybridoma clones, although they stimulated the type II NKT cell hybridoma more strongly than did the type I NKT cell hybridoma. It is not clear why the analogs with a phytosphingosine base group activated type I NKT cells to a limited extent. Clearly, the way the sphingosine and acyl chains fit into pockets in the CD1d molecule can affect T cell specificity even though these are not exposed, but they could influence the orientation of the exposed portion. KRN7000 also has a phytosphingosine base, but it is difficult to make a structural connection without crystallography or other molecular imaging techniques. In contrast, C24:2 with a sphingosine base, as well as C24:1, activated only the type II NKT cell hybridoma, but not the type I NKT cell hybridoma. Therefore, these analogs are type II NKT cell specific. C24:0 activated neither of the hybridoma clones. We also confirmed that the stimulation by sulfatide analogs was CD1d dependent because the presence of anti-CD1d antibody (clone 20H2) completely abolished the reactivity of the sulfatide analogs. In contrast, stimulation by the anti-CD3 antibody (Figure 2A) was not affected. Since C24:1 and C24:2 were specific ligands for the type II NKT cell hybridoma, but not stimulatory for the type I NKT cell hybridoma DN32, we decided to focus on them for further investigation.

C24:1 and C24:2 were titrated with the type I NKT cell hybridoma clone DN32. Neither of these compounds stimulated this type I NKT cell hybridoma at any concentration ranging from 0.1 to 30 μM presented by the CD1d monomer on plastic (Figure 2B, left). At the same time, we again confirmed that C24:1 and C24:2 stimulated the type II NKT cell hybridoma clone XV19 with a bell-shaped titration curve (Figure 2B, right). In addition, we stimulated 2 other type I NKT cell hybridomas, 24.9E and 24.8A, with sulfatide analogs to confirm that these analogs do not stimulate at least 3 different hybridoma clones of type I NKT cells. 24.9E and 24.8A are type I NKT cell hybridoma clones that differ from DN32 by their Vβ/Jβ gene rearrangements (DN32: Vβ8.2/Jβ2.4, 24.9E: Vβ8.3/Jβ2.4, and 24.8A: Vβ8.2/Jβ2.5). They react to different kinds of ligands (e.g., 24.8A reacts more with phosphatidyl-inositol than with αGalCer) and have different magnitudes of reactivity even to the same ligand (31). As shown in Figure 2C, C24:1 and C24:2 did not stimulate either 24.9E or 24.8A. Although KRN7000 did not stimulate 24.8A either, this is consistent with a previous report (31). Thus, these sulfatide analogs presented by CD1d monomers on plastic did not stimulate any of 3 type I NKT cell hybridoma clones we tested.

### The effects of sulfatide analogs on tumor immunity.

**Figure 3:**
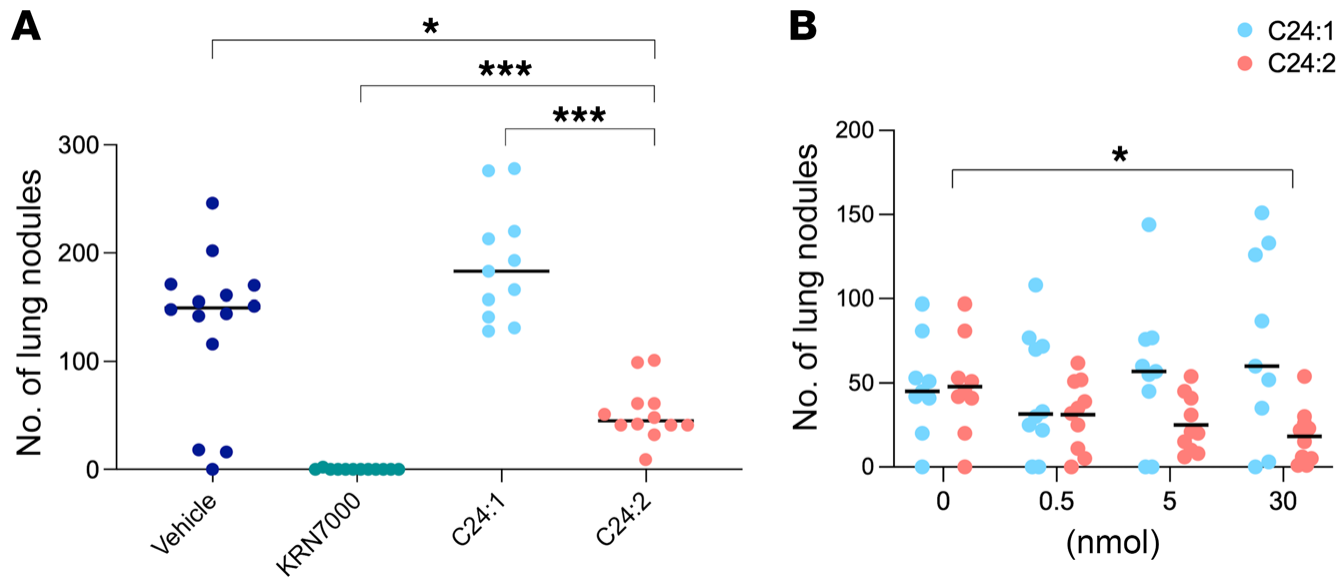
The effects of sulfatide analogs on tumor immunity. (**A**) WT mice were injected i.v. via the tail vein with 5 × 10^5^ CT26 cells and subsequently injected i.p. with the vehicle used to dissolve the sulfatide analogs, 500 pmol KRN7000, or 30 nmol sulfatide analogs. Mice were sacrificed 12 days after tumor challenge, and lung metastases were enumerated. The mean nodule number for each group is indicated by a horizontal bar, and each symbol represents an individual mouse. (**B**) WT mice were injected i.v. into the tail vein with 5 × 10^5^ CT26 cells and subsequently injected i.p. with a graded dose of sulfatide analogs (ranging from 0 to 30 nmol). Mice were sacrificed 14 days after tumor challenge, and lung metastases were enumerated (*n* = 9–10 mice per group). Data represent at least 2 experiments and the mean ± SD. *P < 0.05 and ***P < 0.001. Mann-Whitney test was used to determine *P* values.

We previously reported that in vivo injection of native sulfatide in a murine model of lung metastasis increased the number of lung nodules, whereas KRN7000 reduced it (15). Using the same model, we tested the effect of the sulfatide analogs on the establishment of lung metastases. CT26 tumor cells were injected i.v. into WT mice, which subsequently received a single i.p. injection of lipid. C24:1, which has been reported to be the immunodominant component of native sulfatide, had no significant effect on the number of lung metastases in these WT mice compared with the vehicle-injected group, but C24:2 significantly reduced the development of lung metastases in a dose-dependent manner (Figure 3, A and B), albeit not as completely as KRN7000 did. The unexpected protection by C24:2 could theoretically have been due either to stimulation of an altered functional response of type II NKT cells or stimulation of type I NKT cells.

### C24:2 promotes tumor immunity through an IFN-γ–dependent mechanism.

**Figure 4:**
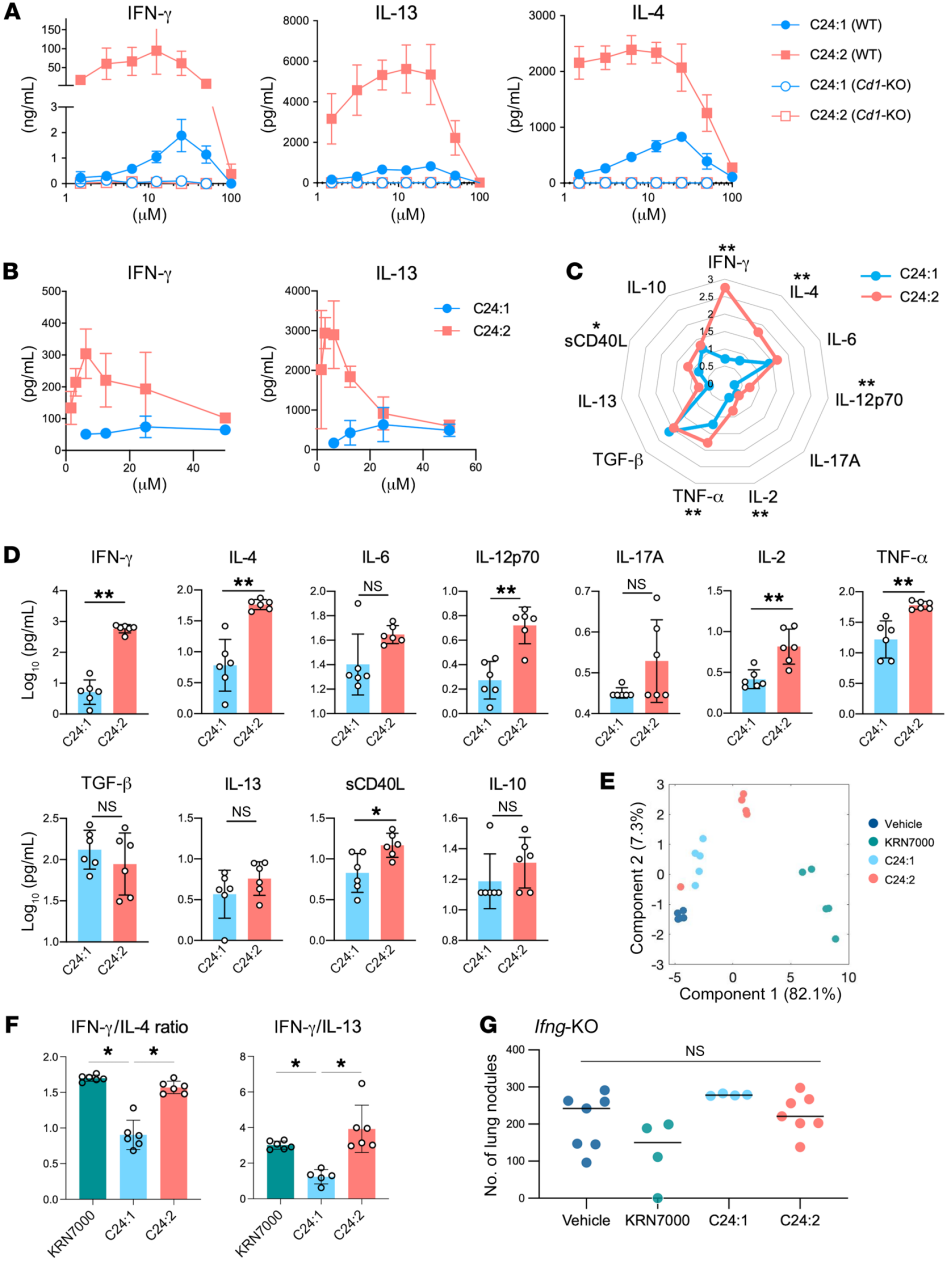
Tumor protection induced by C24:2 is IFN-γ dependent. (**A**) In vitro cytokine response of splenic MNCs from naive WT mice or *Cd1*-KO mice stimulated for 72 hours with a graded dose of sulfatide analogs (mean ± SD of triplicate cultures). (**B**) In vitro cytokine response of lung MNCs from naive WT mice stimulated for 96 hours with BMDCs prepulsed with a graded dose of sulfatide analogs (mean ± SD of triplicate cultures). (**C** and **D**) Radar plot (**C**) and bar graph (**D**) show plasma cytokine levels of mice injected i.p. with 30 nmol sulfatide analogs. Plasma samples were collected 12 hours after lipid injection and analyzed. *n* = 6 mice per group. (**E**) Clustering analysis by PCA of serum cytokine profiles for mice injected i.p. with the vehicle used to dissolve the sulfatide analogs, 500 pmol KRN7000, or 30 nmol sulfatide analogs. Serum samples were collected 3 hours, 6 hours, 12 hours, and 24 hours after lipid injection and analyzed. *n* = 5 mice per group. (**F**) Plasma cytokine levels in mice injected i.p. with 500 pmol KRN7000, 30 nmol C24:1, or 30 nmol C24:2 are plotted as a ratio between IFN-γ and IL-4 (left) or IFN-γ and IL-13 (right). Plasma samples were collected 12 hours after the lipid injection and analyzed. *n* = 6 mice per group. (**G**) *Ifng*-KO mice were injected i.v. via the tail vein with 5 × 10^5^ CT26 cells and subsequently injected i.p. with the vehicle used to dissolve the sulfatide analogs, 500 pmol KRN7000, or 30 nmol sulfatide analogs. Mice were sacrificed 14 days after tumor challenge, and lung metastases were enumerated. The mean nodule number for each group is indicated by a horizontal bar. Each symbol represents an individual mouse. Data represent at least 2 experiments. *P < 0.05 and **P < 0.005 across all groups. Results for **D** were assessed by Mann-Whitney test and results for **E** were assessed by Mann-Whitney U test.

To further investigate the mechanism explaining the different outcomes with C24:1 and C24:2 in vivo, we conducted an ex vivo study. We isolated splenic mononuclear cells (MNCs) from WT mice, stimulated with each sulfatide analog and analyzed cytokine production. Splenic MNCs from WT mice stimulated with C24:2 produced a greater amount of both Th1 and Th2 cytokines compared with C24:1, and this cytokine production was CD1/NKT cell dependent, as splenic MNCs from *Cd1*-deficient (*Cd1*-KO) mice produced no cytokines when stimulated with either lipid (Figure 4A). In addition, we examined lung MNCs, as the lung is the site at which the i.v.-injected CT26 cells are trapped and form the tumor nodules. Similar to the results of the splenic cells, lung MNCs incubated with C24:2-pulsed bone marrow–derived DCs (BMDCs) produced a higher amount of both Th1 and Th2 cytokines compared with lung MNCs stimulated with C24:1-pulsed BMDCs (Figure 4B). We also confirmed in vivo that injection of C24:2 stimulated a much greater amount of cytokine production, as measured in plasma, than did C24:1 in WT mice (Figure 4, C and D). Thus, the increased cytokine production was not limited to an in vitro observation. In addition, we analyzed the serum cytokine levels of mice injected with each lipid across different time points (3 h, 6 h, 12 h, and 24 h after injection). The heatmap of individual cytokine levels in serum showed a pattern similar to that seen in plasma (Supplemental Figure 1; supplemental material available online with this article; https://doi.org/10.1172/JCI165281DS1). The principal component analysis (PCA) data generated from the same experiment demonstrated that the clusters of KRN7000-, C24:1-, and C24:2-injected mice were distinct from each other (Figure 4E). As the cytokine production in plasma stimulated with C24:2 was more Th1 skewed than that stimulated with C24:1, because both ratios of IFN-γ/IL-4 and IFN-γ/IL-13 were higher in C24:2 (Figure 4F), we hypothesized that the difference between the effects of C24:1 and C24:2 in tumor immunity was dependent on IFN-γ production. To address this, we injected each lipid into IFN-γ–deficient (*Ifng*-KO) mice that had been previously injected with CT26 cells and counted lung metastasis nodules. Consistent with our hypothesis, in the absence of IFN-γ, C24:2 did not reduce the number of lung metastases (Figure 4G, no statistically significant differences across all groups), in contrast to that seen in WT mice (Figure 3A). These data suggest that C24:2 promoted tumor immunity through an IFN-γ–dependent mechanism.

Since we observed antitumor function from C24:2 in the CT26 tumor metastasis mouse model, we further verified that C24:2 was not potentially contaminated with a variant form that would be more immunostimulatory, such as the α-anomer of C24:2. To address this, we generated the α-anomer of C24:2 (SR-22-24A) and the α-anomer of C24:1 (C24:1) (Supplemental Figure 3A). We titrated SR-22-24A and C24:2 with BMDCs to activate DN32 and observed a 1,000-fold greater reactivity to the α-anomer (Supplemental Figure 3B). Additionally, we titrated SR-22-24A and C24:1 with CD1d-lipid complexes on plastic and compared them with their β-anomers and observed differing reactivity to DN32 activation (Supplemental Figure 3C). Although we could not assess the magnitude of the difference in reactivity for C24:2 compounds because the β-anomer did not stimulate, we observed that C24:1 had at least a 1,000-fold difference in reactivity when compared with C24:1. Since these compounds all underwent similar synthesis processes, we believe that any potential α-anomer contamination would have to be under 0.1%, or it would have been detected in the hybridoma assays without processing.

### The difference of antigen-presenting cells and cosignaling molecules expressed on APCs between C24:1 and C24:2 injected mice.

**Figure 5:**
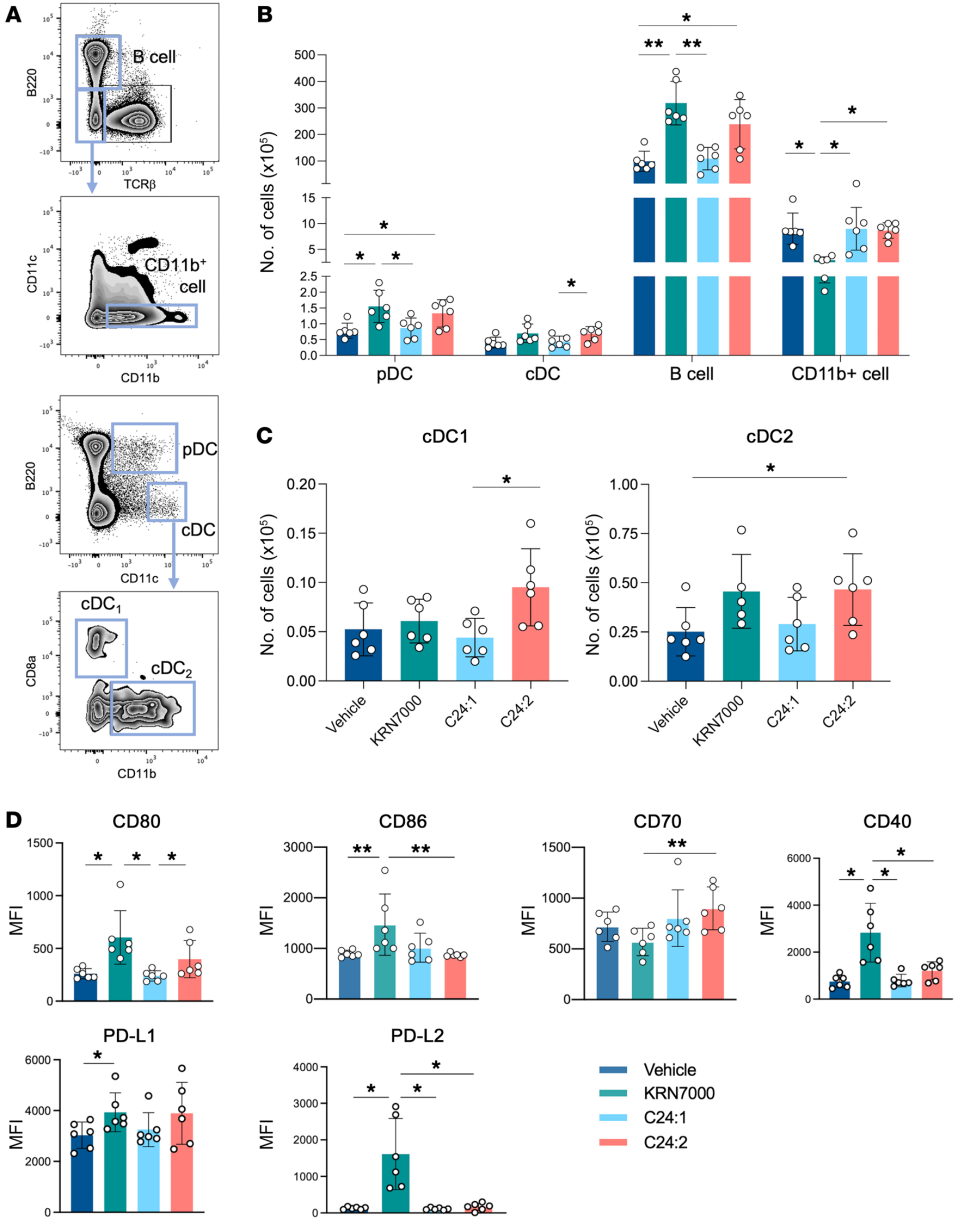
C24:2 induces expansion of cDC, especially cDC1, and higher expression of the costimulating molecule. Mice were injected i.p. with the vehicle used to dissolve the sulfatide analogs, 500 pmol KRN7000, or 30 nmol sulfatide analogs, and spleens were harvested 24 hours later. After staining with mAbs specific for leukocyte markers, flow cytometry was used to gate each indicated cell type. (**A**) Multiparameter staining for cell-type–specific markers and gating strategy for each cell population. (**B** and **C**) Absolute cell numbers of the indicated cells. (**D**) Splenic cDC1s were analyzed by flow cytometry for the indicated cell-surface molecules. Data shown are the mean ± SD. *n* = 6 mice per group. *P < 0.05 and **P < 0.005.

In our in vivo experiment, plasma cytokine production in mice injected with sulfatide analogs showed that the levels not only of IFN-γ but also of other cytokines, including IL-12p70 and sCD40L, were significantly higher in C24:2-injected mice compared with levels in C24:1-injected mice (Figure 4, C and D). These results indicate that the interaction between NKT cells and antigen-presenting cells (APCs) might be involved in the difference in tumor immunity outcomes between C24:1 and C24:2. NKT cells are activated through the recognition of glycolipids presented by CD1d-expressing APCs without affecting their CD1d expression level (Supplemental Figure 2). Also, further outcomes of cellular interactions during immune responses are controlled by cosignaling molecules expressed on the cell surface of APCs. Therefore, to gain more insight into how C24:2 promotes tumor immunity through an IFN-γ–dependent mechanism, we investigated the difference in phenotypes of APCs and their cosignaling molecules between the mice injected with C24:1 and those injected with C24:2. We injected WT mice with each lipid, and after 24 hours, we harvested and analyzed their splenic MNCs stained with mAbs specific for cell-surface markers and cosignaling molecules. As shown in Figure 5B, the number of conventional DCs (cDCs) (CD11c^+^B220^–^, gating shown in Figure 5A) was significantly greater in C24:2-injected mice than in C24:1-injected mice, whereas other APCs (plasmacytoid DCs [pDCs], B220^+^CD11c^+^; B cells, B220^+^ TCRβ^–^; CD11b^+^ cells, B220^–^TCRβ^–^CD11c^–^CD11b^+^ ; Figure 5A) showed no significant differences between mice injected with C24:1 or C24:2. Since there were mainly 2 subsets within cDCs, termed cDC1 and cDC2, we next investigated these 2 subsets. The CD8α^+^CD11b^–^ cell population, which represents cDC1s (cross-presenting DCs), has been reported to be important in antitumor immune responses (32–34). We found that within the cDCs, this cDC1 population was significantly increased in C24:2-injected mice compared with C24:1-injected mice (Figure 5C), although a trend was also seen for more cDC2s. Moreover, among the cosignaling molecules of cDC1s, the MFI of CD80 was significantly higher in C24:2-injected mice than in C24:1-injected mice (Figure 5D). In contrast, it is worth noting that C24:1 seemed to induce little or no changes in APC populations. These results suggest that C24:2, not C24:1, induced cDC (especially cDC1) expansion and higher expression of costimulating molecules and, therefore, induced subsequent immune responses, which resulted in the enhancement of antitumor immunity. These cells may be the source of higher levels of IL-12, which could in turn induce higher levels of IFN-γ.

### Sulfatide-pulsed BMDCs stimulate the type I NKT cell hybridoma, but not the type II NKT cell hybridoma, and are dependent on lysosomal acidification.

**Figure 6:**
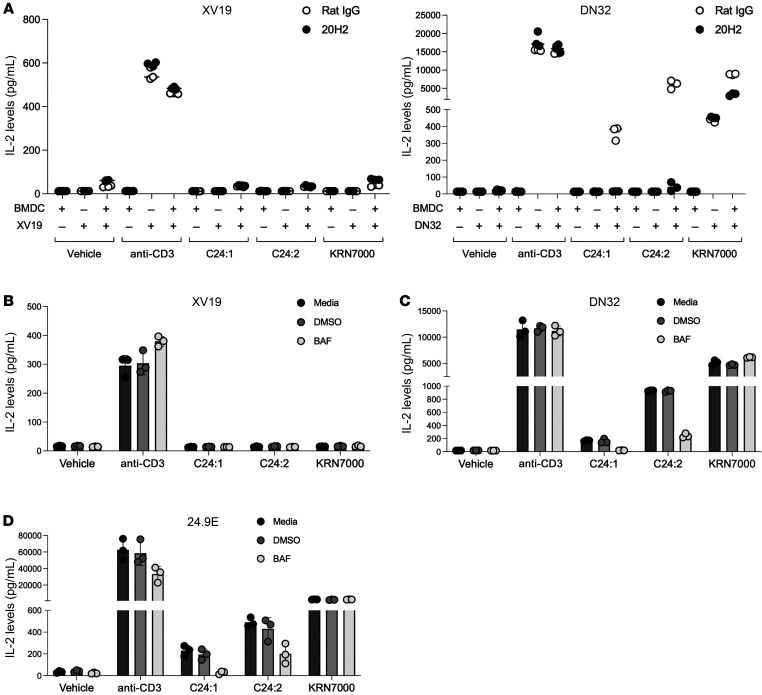
Sulfatide analogs stimulate type I NKT cells but lose their ability to stimulate type II NKT cells when presented by BMDCs. The reactivity of type I or type II NKT cells stimulated with BMDCs prepulsed with sulfatide analogs was evaluated by ELISA to determine IL-2 levels in the culture media. (**A**) The type II NKT hybridoma clone XV19 (left) and the type I NKT hybridoma clone DN32 (right) were stimulated for 16 hours with BMDCs prepulsed with sulfatide analogs (10 μM) or KRN7000 (25 nM), or with 0.25 μg plate-bound anti-CD3 antibody in the presence of 10 μg/mL rat IgG or anti-CD1d antibody (20H2). Data indicate the mean ± SD of triplicate cultures. (**B**–**D**) The type II NKT hybridoma clone XV19 (**B**) and the type I NKT hybridoma clones DN32 (**C**) and 24.9E (**D**) were stimulated for 16 hours with BMDCs prepulsed with sulfatide analogs (10 μM) or KRN7000 (25 nM), or with 0.25 μg plate-bound anti-CD3 antibody in the presence of culture media, 0.03% DMSO, or 50 nM bafilomycin A1 (BAF). Data represent 2 experiments and the mean ± SD of triplicate cultures.

Since we showed that in vivo C24:2 injection induced expansion and activation of cDC1 cells, we next stimulated hybridoma clones using BMDCs as APCs to present sulfatide analogs in vitro. We pulsed BMDCs with each lipid, washed and incubated them with hybridoma clones, and measured IL-2 production in the culture media. IL-2 levels produced by XV19 using BMDCs pulsed with the vehicle, C24:1, C24:2, or KRN7000 were of the same background level (Figure 6A, left), indicating a lack of specific response to the lipids. On the other hand, DN32 was stimulated by C24:1-, C24:2-, or KRN7000-pulsed BMDCs, and those stimulations were CD1d dependent, as the stimulation was blocked by the anti-CD1d antibody 20H2 (Figure 6A, right). In direct contrast to the results of the assays involving stimulation with cell-free CD1d monomer loaded onto the plates shown in Figure 2, these results showed that sulfatide analogs pulsed on BMDCs stimulated the type I NKT cell hybridoma but not the type II NKT cell hybridoma.

It is known that sulfatides are hydrolyzed to cleave the sulfate moiety in lysosomes and become the corresponding β-galactosylceramides (βGalCers) (35). Thus, we speculated that BMDCs internalize the sulfatides, which are then processed and degraded by lysosomal enzymes into βGalCers, which in turn stimulate type I NKT cells. To test that hypothesis, we used the lysosomal acidification inhibitor bafilomycin A1 to inhibit the degradation of sulfatide in lysosomes. As shown in Figure 6C, bafilomycin A1 markedly reduced IL-2 production by DN32 cells stimulated with sulfatide-pulsed BMDCs. We observed a similar pattern in 24.9E cells, another type I hybridoma clone (Figure 6D). These results suggest that the degradation in lysosomes was necessary for sulfatides to stimulate the type I NKT cell hybridoma and that degraded sulfatides did not stimulate the type II NKT cell hybridoma (Figure 6B). The fact that the bafilomycin A1 inhibition of lysosomal processing did not restore stimulation of the type II NKT cell hybridoma (Figure 6B) suggests that the loading of sulfatide takes place primarily in acidified lysosomes. Indeed, we and others have previously observed that loading sulfatide onto free CD1d monomers in vitro requires low pH and lipid transfer proteins as a catalyst (36). Hence, bafilomycin A1 also prevented this loading of unprocessed sulfatide, which required endosomal loading but not surface loading at a neutral pH. Note that bafilomycin A1 did not inhibit stimulation by anti-CD3 as a control for nonspecific inhibition or toxicity.

### βGalCer analogs that result from sulfatase cleavage of the corresponding sulfatides are more potent type I NKT cell stimulators.

**Figure 7:**
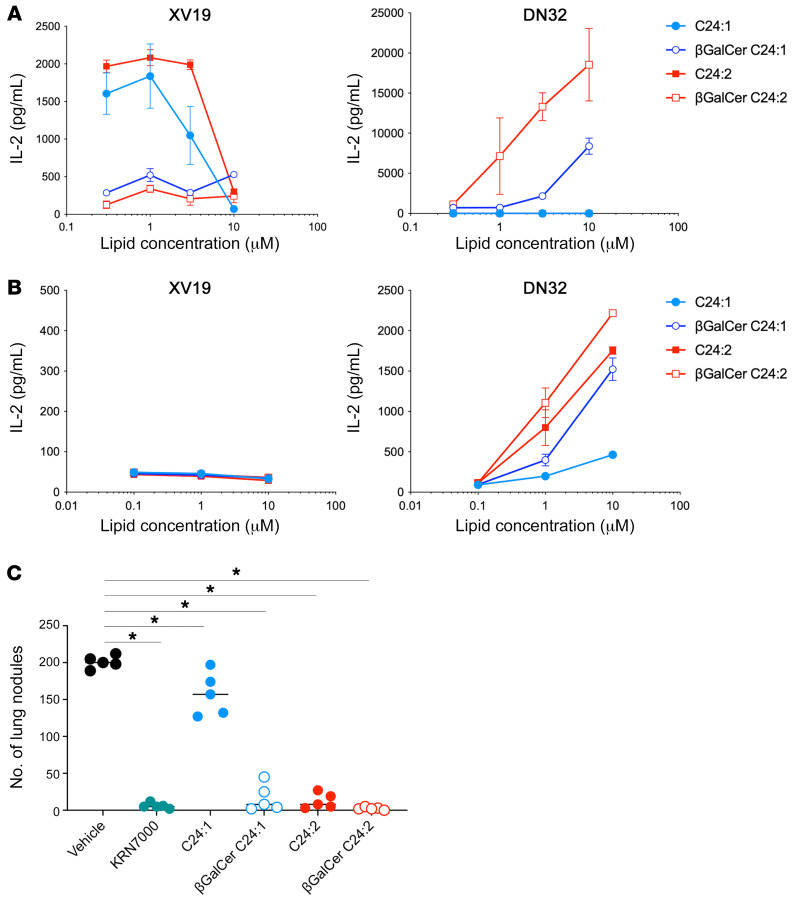
βGalCer stimulates type I NKT cells more potently than the corresponding sulfatides. (**A**) The type II NKT hybridoma clone XV19 (left) and the type I NKT hybridoma DN32 (right) were stimulated for 24 hours with 0.5 μg plate-bound mCD1d monomers loaded with graded concentrations of sulfatide C24:1, sulfatide C24:2, βGalCer C24:1, or βGalCer C24:2. (**B**) The type II NKT hybridoma clone XV19 (left) and the type I NKT hybridoma clone DN32 (right) were stimulated for 16 hours with BMDCs prepulsed with a graded dose of sulfatide analogs or βGalCer analogs. Data indicate the mean ± SD of duplicate cultures. (**C**) WT mice were injected i.v. via tail vein with 5 × 10^5^ CT26 cells and subsequently injected i.p. with the vehicle used to dissolve the sulfatide or βGalCer analogs, 500 pmol KRN7000, or 30 nmol sulfatide or βGalCer analogs. Mice were sacrificed 12 days after tumor challenge, and lung metastases were enumerated. The mean nodule number for each group is indicated by a horizontal bar, and each symbol represents an individual mouse. Data were assessed by Mann-Whitney U test with Holm-Sidak corrections. *P < 0.05. Data represent at least 2 experiments and the mean ± SD.

Although β-linked GalCer analogs have not been found to be very strong stimulators of type I NKT cells or to have potent antitumor activity (37), these βGalCer C24:1 and βGalCer C24:2 analogs had never been tested to our knowledge. We therefore compared sulfatides and their corresponding βGalCer versions both in vitro and in vivo. Figure 7A shows that the plate-bound CD1d monomer loaded with sulfatides stimulated only the type II NKT cell hybridoma, as seen in Figure 2, and that the plate-bound CD1d loaded with βGalCers stimulated only the type I NKT cell hybridoma. On the other hand, Figure 7B shows that BMDCs pulsed with either sulfatides or βGalCers failed to stimulate the type II NKT cell hybridoma, but that both sulfatides and βGalCers stimulated the type I NKT cell hybridoma. Figure 7B also shows that when the lipids were pulsed onto BMDCs and presented to the type I NKT cell hybridoma at the same concentration, IL-2 production by βGalCers was higher than that observed with the corresponding sulfatides. To rule out contamination of the βGalCers with the corresponding α-anomers, we synthesized and tested βGalCers. Supplemental Figure 3B shows that the α-anomer of βGalCer C24:2 was approximately 100-fold more potent on a molar basis for stimulation of DN32, but approximately 10-fold less potent than KRN7000. It is not possible that contamination with KRN7000 could have occurred, as it was not even present in the laboratory that did the synthesis. Chemical tests are not sensitive enough to exclude a 0.1% contamination of βGalCer C24:2 with its α-anomer, but the synthetic method used makes such a contamination extremely unlikely. In Supplemental Figure 3C, we also show a titration comparing the sulfatides C24:1 and C24:2 with their α-anomers (with sulfate) to stimulate DN32 when coated onto CD1d monomers on plastic. Since the α-anomers, but not the β-anomers, stimulated at the highest concentration testable, we think these sulfatides could not have been contaminated with their α-anomers either. The same trends were observed in an in vivo mouse tumor challenge experiment (Figure 7C).

**Figure 8:**
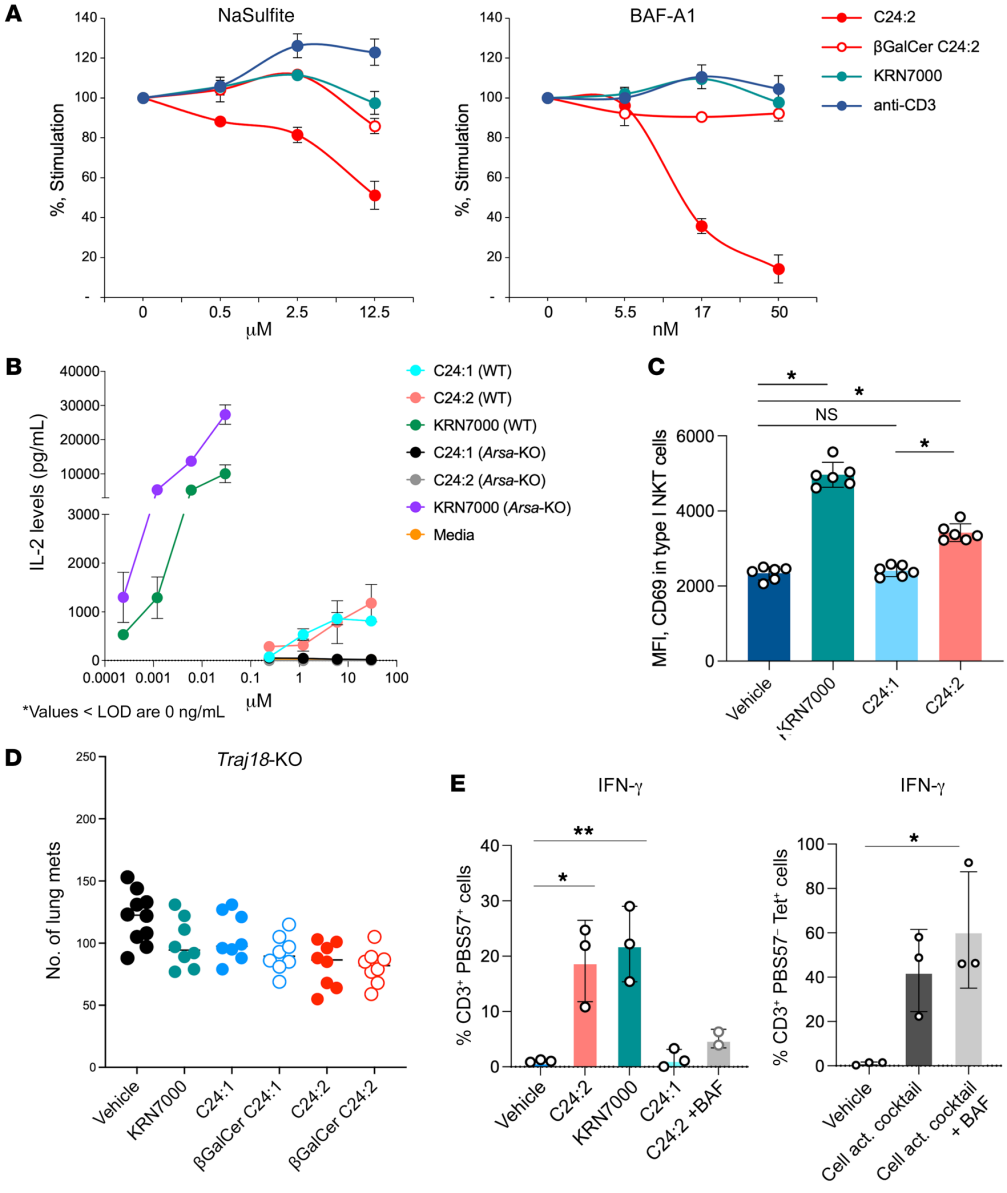
Inhibition of arylsulfatase A prevents the processing of sulfatides, and their antitumor activity is dependent on type I NKT cells. (**A**) Type I NKT hybridoma clone DN32 cells were stimulated with BMDCs prepulsed with either sulfatide C24:2 (10 μM), βGalCer C24:2 (10 μM), or KRN7000 (25 nM), or with 0.25 μg plate-bound anti-CD3 in the presence of titrated 0–12.5 μM NaSulfite (left) or titrated 0–50 nM bafilomycin A1 (BAF-A1) (right) for 16 hours. IL-2 levels in the culture media were measured by ELISA and the percentage and stimulation of each treatment were calculated relative to IL-2 levels in DN32 cells cocultured with PBS-treated BMDCs. Data represent at least 2 experiments and indicate the mean ± SD of duplicate cultures. (**B**) DN32 cells were stimulated for 24 hours with 50,000 BMDCs at a 1:1 ratio. *Arsa*-KO and WT BMDCs were prepulsed with sulfatide analogs, KRN7000, or C24:1. Results are representative data from 2 experiments and indicate the mean ± SD. LOD, limit of detection. (**C**) Mice were injected i.p. with the vehicle used to dissolve the sulfatide analogs, 500 pmol KRN7000, or 30 nmol sulfatide analogs, and spleens were harvested 12 hours later to stain type I NKT cells (CD45^+^, TCRβ^+^, PBS57-loaded CD1d tetramer^+^) and their activation (CD69, MFI) by flow cytometry. Each symbol represents an individual mouse. Data represent at least 2 experiments and the mean ± SD. Data were assessed by the Mann-Whitney U test with Holm-Sidak corrections. (**D**) *Traj18*-KO mice were injected i.v. via the tail vein with 5 × 10^5^ CT26 cells and were subsequently injected i.p. with the vehicle used to dissolve the sulfatide or βGalCer analogs, 500 pmol KRN7000, or 30 nmol sulfatide or βGalCer analogs. Mice were sacrificed 12 days after tumor challenge, and lung metastases were enumerated. The mean nodule number for each group is indicated by a horizontal bar, and each symbol represents an individual mouse. (**E**) Healthy human PBMCs (1 × 10^6^) were cultured with 10 μg/mL glycolipid (C24:2 with and without BAF 50 nM) for 15 hours and then for 1 hour with brefeldin A. Additionally, human PBMCs were cultured with cell activation (act.) cocktail in the presence of BAF-A1. (**E**) Quantification of IFN-γ^+^ type I NKT cells after glycolipid treatment. Data were pooled from 3 experiments and represent the mean ± SD. *P < 0.05 and **P < 0.01, by 2-way ANOVA with Dunnett’s multiple comparisons for batch effects.

To further confirm that the processing of C24:2 sulfatide in DC lysosomes was mediated by the known enzyme arylsulfatase A to cleave the sulfate moiety, we examined the effect of sulfite inhibition of arylsulfatase A, as has been previously described (38). The results showed that titrated amounts of sulfite reduced the stimulation of DN32 by DCs pulsed with C24:2 in a dose-dependent manner, without affecting stimulation by anti-CD3 and KRN7000, as a control for nonspecific toxicity within this concentration range (Figure 8A, left). Moreover, stimulation by βGalCer C24:2 was not affected by the inhibition of arylsulfatase A, as the presentation of βGalCer C24:2 does not require arylsulfatase A. A parallel titration of bafilomycin A1 was also carried out (Figure 8A, right) and showed a similar specific inhibition of stimulation by C24:2 without any effect on KRN7000, βGalCer C24:2, or anti-CD3, as a control for nonspecific toxicity or other inhibitory effects over this concentration range. In addition, to confirm the role of arylsulfatase A in BMDCs in processing C24:2, we used arylsulfatase A–deficient mice (*Arsa*-KO). Indeed, BMDCs generated from *Arsa*-KO mice failed to process and present C24:2 and activate DN32 cells compared with BMDCs generated from WT mice (Figure 8B).

We also hypothesized that if cleavage of βGalCer C24:2 was the mechanism by which DCs pulsed with C24:2 stimulate type I NKT cells, then we should detect activation of type I NKT cells in vivo, and the protection should be dependent on type I NKT cells as well. To test this, we first looked at the induction of expression of the early activation marker CD69 on PBS57-loaded, CD1d-tetramer^+^ type I NKT cells 12 hours after injection of C24:2 in vivo (Figure 8C). Indeed, CD69 expression was significantly upregulated on type I NKT cells compared with the vehicle control and the C24:1 sulfatide, albeit not as significantly as the CD69 expression induced by KRN7000. To test dependence on type I NKT cells, we used BALB/c TCRα joining 18–deficient (*Traj18*-KO) mice, which lack type I NKT cells but retain type II NKT cells. As predicted, no tumor protection was induced by C24:2 in *Traj18*-KO mice (Figure 8D; note that the absence of type I NKT cells in *Traj18*-KO mice did not affect CD1d expression) (39).

These results were further confirmed and shown to be translatable to humans in primary human PBMCs. C24:2 stimulation of human PBMCs induced the production of IFN-γ in type I NKT cells defined as CD3^+^ PBS57-loaded CD1d tetramer^+^ (Supplemental Figure 4 and Figure 8E). Note that the stimulation of human type I NKT cells by C24:2 was inhibited by bafilomycin A1, which shows that the processing we described in the mouse translated to human NKT cells as well. This concentration of bafilomycin A1 was not toxic to human NKT cells, as we show in the right panel of Figure 8E that it did not affect their activation by cell activation cocktail (PMA and ionomycin), which does not require processing.

We conclude that tumor protection was mediated by the stimulation of protective type I NKT cells by the processed sulfatide glycolipid, a phenomenon not previously described to our knowledge. This basic phenomenon of processing sulfatides in endosomes to change the cellular specificity of the lipid translated to human type I NKT cells as well.

## Discussion

In this study, we demonstrated that the newly synthesized sulfatide analog C24:2 had a potent antitumor effect induced through an IFN-γ–dependent mechanism, distinct from the biological effect of C24:1 that differs by only 1 double bond. We found, unexpectedly, that when the sulfatide analogs C24:1 and C24:2 were presented by DCs, they lost their ability to stimulate a type II NKT cell hybridoma and gained the ability to stimulate several type I NKT cell hybridomas. This was abrogated by bafilomycin A1, which blocks endosomal/lysosomal processing, and by blockade (or KO) of arylsulfatase A, which can cleave the sulfate moiety, suggesting that the tumor protection afforded by C24:2 in vivo may depend not on altered stimulation of type II NKT cells but on altered specificity for different target cells caused by lysosomal processing to stimulate type I NKT cells, in addition to inducing the activation and expansion of cDC1 cells.

Yu et al. previously reported that altering the ceramide structure of KRN7000 by having a shortened, unsaturated fatty acid chain or having a sphingosine base instead of a phytosphingosine base could modify the functional property of type I NKT cells (20). However, similar structure-function studies have not been available for type II NKT cell ligands. We sought to investigate whether altered structures of type II NKT cell ligands might produce altered functions that could potentially be used to overcome their immunosuppressive activity and develop new therapeutic strategies for cancer immunotherapies. Our study demonstrated that, indeed, the immunoreactivity of type II NKT cells was also modified by altering the number of double bonds in a fatty acid chain or of a sphingoid base group in the ceramide structure of sulfatide. Previous studies (27, 28) showed that, among sulfatide isoforms that contain a fatty acid chain, C24:1 induces the most potent stimulation of a type II NKT cell hybridoma. Consistent with these data, our results also demonstrated that C24:1 induced the highest immunoreactivity of the type II NKT cell hybridoma among the 6 sulfatide analogs we tested, of which 4 were newly synthesized analogs. However, such titrations cannot distinguish the affinity for CD1d from the affinity of the TCR for the CD1d-lipid complex. One of the strengths of our study is that we also investigated the immunoreactivity of each sulfatide analog by both type I and type II NKT cells simultaneously presented either on a CD1d monomer on plastic or pulsed onto BMDCs. We confirmed that analogs with a sphingosine base stimulated type II NKT cells specifically and exclusively when presented on CD1d on plastic. In contrast, analogs with a phytosphingosine base stimulated both type I (albeit to a substantially lesser extent) and type II NKT cells. Some stimulation of type I NKT cells by sulfatides with a phytosphingosine base has been described before (40). To provide more rigorous evidence that the specificity of the sphingosine base group is for the activation of type II (not type I) NKT cells, we used 3 different type I NKT cell hybridoma clones (DN32, 24.9E, and 24.8A) that have distinct TCRs and different immunoreactivities and fine specificities. We found that C24:1 and C24:2 presented by the CD1d monomer on plastic induced no reactivity from any of these type I NKT cell hybridoma clones. These results suggested that C24:1 and C24:2 (sphingosine base) are both exclusive ligands for type II NKT cells, even though they induced different functional properties of type II NKT cells.

Our current data demonstrated that sulfatide analogs that possessed a phytosphingosine base also stimulated the type II NKT cell hybridoma. The phytosphingosine base group induced immunoreactivity not only by the type II NKT cell hybridoma but also by the type I NKT cell hybridomas. Wu et al. previously reported that a sulfatide analog that possessed a phytosphingosine base was stimulatory for human type I NKT cells (40). This, together with our data, suggests that the alteration of the structure from a sphingosine base to a phytosphingosine base may play a role in the ability of these sulfatide analogs to stimulate type I NKT cells. Further research is needed to elucidate the underlying mechanism for these observations.

Previous reports also showed that sulfatide and its analogs do not stimulate type I NKT cells (10, 12). Previous sulfatide analog studies have mostly involved experiments using hybridomas, so it was not possible to determine whether the different sulfatide isoforms could modulate the cytokine profiles or disease outcomes. Our results above also focused on NKT cell hybridomas and their reactivity to sulfatide analogs presented by CD1d monomers coated onto the plastic culture plates. We developed our study to include ex vivo and in vivo settings so that we could assess the interaction of sulfatide analogs with primary mouse cells and the effects on tumor growth. Surprisingly, a single in vivo injection of C24:2 in a murine model of lung metastasis reduced the development of lung nodules, whereas C24:1 produced no significant difference compared with the control group. We also discovered that C24:2 induced more cytokine production both in vitro and in vivo than did C24:1, and the profile was more skewed toward Th1 cytokines. This skewing toward IFN-γ contributed to protection and was supported by the finding that C24:2 treatment was not effective in reducing lung metastases in *Ifng*-KO mice compared with WT mice.

Our in vivo analysis of plasma cytokines showed that IL-12p70 (*P* < 0.005) and sCD40L (*P* < 0.05), as well as IFN-γ (*P* < 0.005), were significantly elevated in C24:2-injected mice compared with levels in C24:1-injected mice. IL-12 production indicates the involvement of APCs in the differences between C24:1 and C24:2. We analyzed the phenotypes of APCs in mice injected with C24:1 or C24:2 and found that C24:2 induced an increase of cDCs, especially cross-presenting CD8α^+^CD11b^–^ DCs (cDC1s) among APCs. Moreover, the expression of the costimulatory molecule CD80 on cDC1s was significantly (*P* < 0.05) elevated in C24:2-injected mice compared with C24:1-injected mice. These results suggest that C24:2 induced not only the expansion of cDC1s but also the activation of these cells. It is worth noting that C24:2 is best compared with C24:1, as they both require intracellular processing and were also injected at a dose of 30 nmol each, in contrast to KRN7000, which was injected at 500 pmol. Recently, Arora et al. demonstrated a novel concept regarding the different functions between Th1 cell–biasing (α-C-GalCer) and Th2 cell–biasing (C20:2) KRN7000 analogs (23). They showed that all KRN7000 analogs tested (KRN7000, α-C-GalCer, and C20:2) were mainly presented by cDC1s and that qualitative changes in cDC1s contributed to determining the different cytokine profiles induced by KRN7000 analogs (23). cDC1s have a critical role in inducing tumor immunity in vivo (32, 33). They can efficiently process and load exogenously acquired antigens on MHC-I molecules and present to CD8^+^ T cells (41). Moreover, they are the main source of the Th1-polarizing cytokine IL-12 (42, 43), which is involved in the induction of CD4^+^ Th1 responses through upregulation of the Tbet transcription factor. In C24:2-injected mice, the expanded and activated cDC1s might have contributed, possibly through IL-12, to the substantial amount of IFN-γ production, which in turn exerted the antitumor effect observed. However, further studies are needed to elucidate the detailed mechanisms of cDC1s in the tumor immunity in our model.

The potential importance of cDC1 cells led us to ask what the hybridoma response would be like when the sulfatides were presented by DCs rather than by the cell-free CD1d monomer coated onto plastic. To our surprise, DC presentation completely reversed the specificity not only of C24:2 but also of C24:1 sulfatide. Whereas with the cell-free presentation, it stimulated the type II NKT cell hybridoma XV19 exclusively, but none of the 3 different type I hybridoma clones tested (Figure 2), when presented by BMDCs, C24:2 stimulated only the type I NKT cell hybridoma DN32 and no longer stimulated XV19 (Figures 6 and 7). This is consistent with the finding of Blomqvist et al. (27) that C24:1 weakly stimulated the type II NKT cell hybridoma XV19 when presented by RMA-S cells, and stimulated even more weakly when presented by BMDCs, especially when compared with lysosulfatide. This result suggested that the DCs processed the sulfatide into a lipid that stimulated type I, but not type II, NKT cells.

Sulfatides differ from the corresponding βGalCers by the sulfate moiety and from KRN7000 also by the sphingosine rather than the phytosphingosine chain and by double bonds in the acyl chain and its length and by the β-linkage of the sugar. Although such β-linked GalCer structures have not been known to have substantial antitumor activity or to stimulate type I NKT cells very strongly, βGalCer C24:2 had not, to our knowledge, been previously tested. Furthermore, it is known that lysosomes contain the enzyme arylsulfatase A, which can cleave that sulfate moiety (35, 38). Our data showed that bafilomycin A1, which inhibits endosomal/lysosomal acidification and processing, and arylsulfatase A blockade by sulfite both markedly inhibited the ability of C24:2 on BMDCs to stimulate DN32, supporting our hypothesis that the stimulation of type I NKT cells with C24:2 loaded onto DCs is due to endosomal/lysosomal processing by arylsulfatase A to remove the sulfate moiety. Moreover, the βGalCer C24:2 structure could stimulate a type I hybridoma and could protect against the tumor in vivo. This finding itself was novel, in our view, because most βGalCer analogs tested previously did not stimulate type I NKT cells very effectively (44–46), even with a phytosphingosine moiety, and the βGalCer C24:2 analog differed from KRN7000 not only in the β linkage of the galactose but also in the sphingosine chain. However, to our knowledge, this particular βGalCer analog had never been synthesized or tested before and seems to have unusual properties worthy of further investigation.

If our hypothesis is true, then βGalCer C24:2, derived from C24:2, would activate type I NKT cells in vivo, and the protection against tumors by C24:2 would not take place in the absence of type I NKT cells. As predicted, C24:2 did activate type I NKT cells in vivo, and *Traj18*-KO mice failed to be protected against the CT26 tumor. This outcome confirms the surprising result that it was not C24:2 that directly protected by skewing the function of type II NKT cells, but rather it was the processed form of this molecule, βGalCer C24:2, that protected by stimulating type I NKT cells.

We further showed that this basic phenomenon of endosomal processing of C24:2 to allow it to stimulate type I NKT cells translates to human NKT cells as well, as human peripheral blood type I NKT cells (staining with PBS57-loaded CD1d tetramer) were stimulated by C24:2 in the presence of human APCs, and this was inhibited by bafilomycin A1, an endosomal processing inhibitor. This translation greatly increases the potential for applicability to human cancer immunotherapy.

In summary, we have demonstrated that modification of the ceramide portion of sulfatides could alter the function of a type II NKT cell agonist and its downstream effects on tumor growth. Notably, we found the highly unanticipated result that treatment with C24:2 elicited an antitumor effect. The surprise was that the difference was based on antigen processing of the glycolipid rather than a different type II NKT cell activity. We believe this is the first demonstration of DC processing of a glycolipid agonist of NKT cells of either type that reversed its function by altering the specificity for the target cell, in this case from stimulation of type II NKT cells to stimulation of type I NKT cells, which alters the functional activity of the lipid to a protective one. Although some NKT agonist glycolipids may need loading in lysosomes rather than on the cell surface (21, 25), lysosomal processing of NKT cell lipid antigens was an unexpected phenomenon, as antigen processing is known primarily for protein antigens presented by classical MHC molecules to conventional T cells, with rare exceptions such as the GalGal analog of KRN7000 (24). The fact that the processing is not necessary for loading into the MHC-like molecule CD1d, since both C24:2 and its processed form, βGalCer C24:2, can load into the CD1d monomer on plastic, but rather is necessary to alter the specificity for the target cell, we believe is also a previously unrecognized role of antigen processing in itself, as conventional antigen processing of proteins occurs to allow loading onto MHC molecules. This study sheds light on the importance of lysosomal lipid processing, which influences which type of NKT cell is activated and thus alters subsequent immune responses. We propose that these findings may allow the development of new therapeutic targets and agents for cancer immunotherapy.

## Methods

### Mice.

BALB/c mice were purchased from Charles River Laboratories; BALB/c IFN-γ–deficient (*Ifng*-KO), and B6 arylsulfatase A–deficient (*Arsa*-KO) mice were purchased from The Jackson Laboratory; and BALB/c CD1-deficient (*Cd1*-KO) mice (deficient in both the *Cd1d1* and *Cd1d2* genes) were provided by M. Grusby (Harvard University, Boston, Massachusetts, USA). *Traj18*-KO mice were provided by H. Watarai (Kanazawa University, Kanazawa, Japan). All mice were bred at the NCI, NIH. Female mice older than 8 weeks were used in the experiments, and all mice were maintained in a specific pathogen–free animal facility. All possible efforts were made to minimize animal suffering.

### Cell lines.

The type I NKT cell hybridoma clone DN32.D3 was provided by A. Bendelac (University of Chicago, Chicago, Illinois, USA), and 24.9E and 24.8A were gifts from S. Behar (Harvard University, Boston, Massachusetts, USA). The type II NKT cell hybridoma clone XV19 was provided by S. Cardell (University of Gothenburg, Gothenburg, Sweden). All NKT cell hybridoma clones were cultured in RPMI 1640 supplemented with 10% FCS, l-glutamine, sodium pyruvate, nonessential amino acids, streptomycin, penicillin, and 2-mercaptoethanol (0.05 mM). The CT26 colon carcinoma cell line (an *N*-nitro-*N*-methylurethane–induced BALB/c murine colon carcinoma) was provided by N. Restifo (NCI, NIH, Bethesda, Maryland, USA) and maintained in RPMI 1640 supplemented with 10% FCS, l-glutamine, sodium pyruvate, nonessential amino acids, streptomycin, and penicillin.

### Reagents.

Synthetic lipids (KRN7000, C24:0, C24:1, and βGalCer C24:1) were purchased from Avanti Polar Lipids (Alabaster). C24:2, pC24:0, pC24:1, pC24:2, and βGalCer C24:2 were synthesized in our laboratory at the University of Connecticut, as detailed in Supplemental Methods. All lipids were dissolved in the vehicle (0.5% polysorbate-20) and diluted in PBS or complete RPMI 1640 medium with 10% FBS. For the hybridoma assay, sulfatide analogs and βGalCer analogs were dissolved in DMSO (Life Technologies, Thermo Fisher Scientific). The purified anti-CD1D1 antibody (clone 20H2) was purchased from Harlan. Rat IgG was purchased from MilliporeSigma. The anti–mouse CD3 antibody was purchased from BioLegend (clone 145-2C11). Mouse CD1D1 monomers were provided by the NIH Tetramer Core Facility (Emory University, Atlanta, Georgia, USA). The fluorescent protein–labeled mAbs used for flow cytometry are detailed in the Supplemental Methods. Bafilomycin A1 (MilliporeSigma) was dissolved in DMSO and added to cell cultures at a final concentration of 50 nM, 5 minutes before adding lipid antigens. Sodium sulfite (MilliporeSigma) was dissolved in PBS and used at the concentrations shown.

### In vivo lung metastasis assay.

Mice were injected i.v. via the tail vein with a single-cell suspension of 5 × 10^5^ CT26 cells in 200 μL PBS. Subsequently, a single dose of the glycolipids was diluted from the stock solutions at the desired concentration in 100 μL PBS and administered i.p. within 1 hour of tumor challenge. KRN7000 was administered at 5 μM, whereas sulfatide and βGalCer analogs were administered at 300 μM, as previously reported (15, 47). Mice were sacrificed 12 days after tumor cell inoculation, and pulmonary metastases of CT26 cells were enumerated as previously described (48).

### Flow cytometric analysis.

The surface-stained cells were analyzed with a FACSymphony flow cytometer (BD Biosciences), and the data were processed using FlowJo software (version 10.5.2). The following calculation determined the absolute number of cells in each cell subset: total splenic MNC number × the corresponding cell subset proportion to the total CD45^+^ cells.

### BMDCs.

To obtain BMDCs, BM cells were isolated from BALB/c mice and suspended at a concentration of 2 × 10^5^/mL in complete RPMI 1640 medium with 10% FBS in the presence of 20 ng/mL GM-CSF (Peprotech) for 8 days. Fresh medium supplemented with 20 ng/mL GM-CSF was added on day 3 and refreshed on day 6. On day 8 of culturing, cells were harvested, washed in complete medium, and suspended at 4 × 10^5/^mL in complete medium supplemented with 10 ng/mL GM-CSF for 24 hours before being pulsed with glycolipids and used for lung MNC or hybridoma stimulation.

### Plate-bound mCD1d hybridoma stimulation assay.

The protocol was modified from our previous report (47). The mCD1d monomer was incubated at a concentration of 8 μg/mL with the indicated concentrations of sulfatide or βGalCer analogs in pH 5.0 sodium acetate buffer containing 10 μg/mL saposin C (Enzo Life Sciences). After incubation at 37°C overnight, the acidic buffer was replaced with PBS and concentrated using Amicon Ultra Centrifugal Filter Units 30K (MilliporeSigma). Then, 96-well, flat-bottomed plates were coated overnight with 0.5 μg mCD1d monomers loaded with graded concentrations of sulfatide or βGalCer analogs, or 8.73 μM KRN7000, or 10 μg/mL anti–mouse CD3 antibody. Plates were then washed with PBS, and 5 × 10^4^ hybridoma cells in 200 μL complete medium were plated with rat IgG or 20H2. Cells were cultured at 37°C, 5% CO_2_. Supernatants from 24-hour cultures were collected and used in ELISAs to measure secreted IL-2 using the DuoSet kit (R&D Systems) according to the manufacturer’s instructions. The samples were analyzed in duplicate or triplicate.

### Cytokine evaluation assay.

For comparison of the cytokine secretion profiles, a single-cell suspension of splenic MNCs from naive animals was cultured in 96-well, round-bottomed plates (1 × 10^6^ cells/well) in the presence of graded concentrations of glycolipids. Cells were cultured at 37°C, 5% CO_2_, and supernatants were collected at 72 hours to measure cytokine secretion levels using the DuoSet kit. Lung MNCs from naive animals were cultured in 96-well, flat-bottomed plates (2 × 10^5^ cells/well) with BMDCs (4 × 10^4^ cells/well) prepulsed with graded concentrations of glycolipids for 3 hours at 37°C. Cells were cultured at 37°C, 5% CO_2_, and supernatants were collected at 96 hours to measure cytokine secretion levels using the DuoSet kit. To quantify plasma or serum cytokine levels, bead-based multiplex LEGENDplex analysis (BioLegend) was used following the manufacturer’s instructions.

### Intracellular cytokine staining of human NKT cells.

PBMCs were isolated using the Ficoll-Paque density gradient method and frozen until use. Human NKT cells were taken from a healthy donor and cultured in RPMI 1640 supplemented with 10% FCS, l-glutamine, sodium pyruvate, nonessential amino acids, streptomycin, penicillin, and 2-mercaptoethanol (0.05 mM). Human PBMCs were plated on a 96-well plate at 1 × 10^6^ cells per well and stimulated with glycolipid (10 μg/mL), cell activation cocktail (BioLegend), or bafilomycin A1 for 15 hours and subsequently treated with brefeldin A (Invitrogen, Thermo Fisher Scientific) for 1 hour. Samples were stained with the flow cytometric antibodies anti-CD3 (clone SP43-2) and anti–IFN-γ (clone B27) (BD Biosciences), PBS57-loaded CD1d tetramer (NIH Tetramer Core Facility, Atlanta, Georgia, USA), and LIVE/DEAD Fixable Blue Dead Cell Stain (Invitrogen, Thermo Fisher Scientific).

### Statistics.

Data are expressed as the mean ± SD for each group. Statistical differences between groups were evaluated by Mann-Whitney *U* test with Holm-Šidák corrections for multiple comparisons or 2-way ANOVA with Dunnett’s multiple-comparisons for batch effects using GraphPad Prism (version 8.1.1, GraphPad Software). *P* values of less than 0.05 were considered significant. Data are presented as the mean ± SD.

### Study approval.

Animal experiments were conducted in accordance with the protocol approved by the NCI’s Animal Care and Use Committee. Human PBMCs were obtained from the NIH Division of Transfusion Medicine from healthy donors under an IRB-approved NIH protocol (99-CC-0168). Research blood donors provided written informed consent, and blood samples were deidentified prior to distribution (ClinicalTrials.gov NCT00001846).

### Data and materials availability.

The authors confirm that the data supporting the findings of this study are available within the article and in its supplementary material. Values underlying the data presented in each graph and as means are provided in the Supplemental Supporting Data Values file. Data sets analyzed during the current study are available from the corresponding author on request.

## Author contributions

SK, LP, KN, MT, PBO, ARH, and JAB designed the study. KN, LP, KC, JD, SK, AB, SKR, KSH, and TJ collected the data. KN, LP, SK, JAW, JCJ, MT, PBO, KSH, and JAB analyzed the data. KN, LP, KC, JD, SC, SKR, HW, MT, PBO, ARH, KSH, and JAB interpreted the data and discussed the conclusions. KN, LP, KC, JD, and KSH contributed equally to this work. The order of the co–first authors was determined on the basis of the significance, time, and effort each author invested in the project. KN, LP, MT, PBO, ARH, KSH, and JAB wrote the manuscript, which all authors critiqued.

## Supplementary Material

Supplemental data

Supporting data values

## Version 1 12/21/2023

In-Press Preview

## Version 2 02/15/2024

Electronic publication

## References

[1] Sharma P, Allison JP (2015). Immune checkpoint targeting in cancer therapy: toward combination strategies with curative potential. Cell.

[2] Zou W (2016). PD-L1 (B7-H1) and PD-1 pathway blockade for cancer therapy: mechanisms, response biomarkers, and combinations. Sci Transl Med.

[3] Taniguchi M (2003). The regulatory role of Valpha14 NKT cells in innate and acquired immune response. Annu Rev Immunol.

[4] Godfrey DI (2004). NKT cells: what’s in a name?. Nat Rev Immunol.

[5] Kronenberg M (2005). Toward an understanding of NKT cell biology: progress and paradoxes. Annu Rev Immunol.

[6] Bendelac A (2007). The biology of NKT cells. Annu Rev Immunol.

[7] Rossjohn J (2012). Recognition of CD1d-restricted antigens by natural killer T cells. Nat Rev Immunol.

[8] Stetson DB (2003). Constitutive cytokine mRNAs mark natural killer (NK) and NK T cells poised for rapid effector function. J Exp Med.

[9] Cerundolo V (2009). Harnessing invariant NKT cells in vaccination strategies. Nat Rev Immunol.

[10] Jahng A (2004). Prevention of autoimmunity by targeting a distinct, noninvariant CD1d-reactive T cell population reactive to sulfatide. J Exp Med.

[11] Halder RC (2007). Type II NK T cell-mediated anergy induction in type I NK T cells prevents inflammatory liver disease. J Clin Invest.

[12] Subramanian L (2012). NKT cells stimulated by long fatty acyl chain sulfatides significantly reduce the incidence of type 1 diabetes in nonobese diabetic mice [corrected]. PLoS One.

[13] Zhang G (2011). Sulfatide-activated type II NKT cells prevent allergic airway inflammation by inhibiting type I NKT cell function in a mouse model of asthma. Am J Physiol Lung Cell Mol Physiol.

[14] Pan H (2019). Sulfatide-activated type II NKT cells suppress immunogenic maturation of lung dendritic cells in murine models of asthma. Am J Physiol Lung Cell Mol Physiol.

[15] Ambrosino E (2007). Cross-regulation between type I and type II NKT cells in regulating tumor immunity: a new immunoregulatory axis. J Immunol.

[16] Morita M (1995). Structure-activity relationship of alpha-galactosylceramides against B16-bearing mice. J Med Chem.

[17] Kawano T (1997). CD1d-restricted and TCR-mediated activation of valpha14 NKT cells by glycosylceramides. Science.

[18] Miyamoto K, S et al (2001). A synthetic glycolipid prevents autoimmune encephalomyelitis by inducing TH2 bias of natural killer T cells. Nature.

[19] Schmieg J (2003). Superior protection against malaria and melanoma metastases by a C-glycoside analogue of the natural killer T cell ligand alpha-galactosylceramide. J Exp Med.

[20] Yu KO (2005). Modulation of CD1d-restricted NKT cell responses by using N-acyl variants of alpha-galactosylceramides. Proc Natl Acad Sci U S A.

[21] Im JS (2009). Kinetics and cellular site of glycolipid loading control the outcome of natural killer T cell activation. Immunity.

[22] Wu TN (2011). Avidity of CD1d-ligand-receptor ternary complex contributes to T-helper 1 (Th1) polarization and anticancer efficacy. Proc Natl Acad Sci U S A.

[23] Arora P (2014). A single subset of dendritic cells controls the cytokine bias of natural killer T cell responses to diverse glycolipid antigens. Immunity.

[24] Prigozy TI (2001). Glycolipid antigen processing for presentation by CD1d molecules. Science.

[25] Bai L (2009). Lysosomal recycling terminates CD1d-mediated presentation of short and polyunsaturated variants of the NKT cell lipid antigen alphaGalCer. Proc Natl Acad Sci U S A.

[26] Karlsson KA (1970). On the chemistry and occurrence of sphingolipid long-chain bases. Chem Phys Lipids.

[27] Blomqvist M (2009). Multiple tissue-specific isoforms of sulfatide activate CD1d-restricted type II NKT cells. Eur J Immunol.

[28] Zajonc DM (2005). Structural basis for CD1d presentation of a sulfatide derived from myelin and its implications for autoimmunity. J Exp Med.

[29] Maricic I (2014). Dendritic cells and anergic type I NKT cells play a crucial role in sulfatide-mediated immune regulation in experimental autoimmune encephalomyelitis. J Immunol.

[30] Cardell S (1995). CD1-restricted CD4^+^ T cells in major histocompatibility complex class II-deficient mice. J Exp Med.

[31] Gumperz JE (2000). Murine CD1d-restricted T cell recognition of cellular lipids. Immunity.

[32] Hildner K (2008). Batf3 deficiency reveals a critical role for CD8alpha^+^ dendritic cells in cytotoxic T cell immunity. Science.

[33] Hammerich L (2019). Systemic clinical tumor regressions and potentiation of PD1 blockade with in situ vaccination. Nat Med.

[34] Merad M (2013). The dendritic cell lineage: ontogeny and function of dendritic cells and their subsets in the steady state and the inflamed setting. Annu Rev Immunol.

[35] Takahashi T, Suzuki T (2012). Role of sulfatide in normal and pathological cells and tissues. J Lipid Res.

[36] Zhou D (2004). Editing of CD1d-bound lipid antigens by endosomal lipid transfer proteins. Science.

[37] Parekh VV (2004). Quantitative and qualitative differences in the in vivo response of NKT cells to distinct alpha- and beta-anomeric glycolipids. J Immunol.

[38] Matzner U (2009). Saposin B-dependent reconstitution of arylsulfatase A activity in vitro and in cell culture models of metachromatic leukodystrophy. J Biol Chem.

[39] Lang ML (2009). How do natural killer T cells help B cells?. Expert Rev Vaccines.

[40] Wu D (2005). Bacterial glycolipids and analogs as antigens for CD1d-restricted NKT cells. Proc Natl Acad Sci U S A.

[41] Den Haan JM (2000). CD8(+) but not CD8(-) dendritic cells cross-prime cytotoxic T cells in vivo. J Exp Med.

[42] Maldonado-Lopez R (1999). CD8alpha^+^ and CD8alpha^–^ subclasses of dendritic cells direct the development of distinct T helper cells in vivo. J Exp Med.

[43] Hochrein H (2001). Differential production of IL-12, IFN-alpha, and IFN-gamma by mouse dendritic cell subsets. J Immunol.

[44] Parekh VV (2004). Quantitative and qualitative differences in the in vivo response of NKT cells to distinct alpha- and beta-anomeric glycolipids. J Immunol.

[45] Ilan Y (2009). Alpha versus beta: are we on the way to resolve the mystery as to which is the endogenous ligand for natural killer T cells?. Clin Exp Immunol.

[46] Lalazar G (2009). Beta-glycoglycosphingolipid-induced alterations of the STAT signaling pathways are dependent on CD1d and the lipid raft protein flotillin-2. Am J Pathol.

[47] O’Konek JJ (2011). Mouse and human iNKT cell agonist β-mannosylceramide reveals a distinct mechanism of tumor immunity. J Clin Invest.

[48] Park JM (2004). Unmasking immunosurveillance against a syngeneic colon cancer by elimination of CD4^+^ NKT regulatory cells and IL-13. Int J Cancer.

