# Supplementary material for: Lysosomal processing of sulfatide analogs alters target NKT cell specificity and immune responses in cancer
Source: J Clin Invest. 2023 Dec 21;134(4):e165281. doi: 10.1172/JCI165281 (PMC10866642; doi:10.1172/JCI165281)
Supplement: Supplemental data [file jci-134-165281-s234.pdf]

Supplementary Materials for

Lysosomal processing alters the specificity of sulfatide analogues for NKT cells and subsequent immune responses in cancer

Supplementary Methods

General experimental for synthesis of sulfatide analogues

Tetrahydrofuran (THF) was dried using a solvent dispensing system (SDS) with a column of neutral alumina. Pyridine, toluene, dimethylformamide (DMF), methylene chloride (CH_2Cl_2), deuterated chloroform ($CDCl_3$), methanol (MeOH), deuterated methanol (CD_3OD) and ethanol (EtOH) were dried over 4Å molecular sieves (MS). The other reagents were purchased from Acros, Alfa Aesar, Oakwood or Aldrich and used without further purification.

All reactions were conducted under an atmosphere of N_2 in glassware that had been dried overnight in an oven at 120 °C. Where appropriate, control of the reaction temperature was achieved with a solid CO_2/acetone bath, an ice bath or a heated oil bath.

^{1}H NMR spectra were recorded at 500 MHz or 400 MHz, and chemical shifts are calibrated to the residual $CHCl_3$ peak in $CDCl_3$ at 7.26 ppm, to the TMS peak at 0.0, or to the residual CD_3OH peak in CD_3OD at 3.34 ppm. ^{13}C NMR spectra were recorded at 125 MHz or 100 MHz and calibrated to the residual $CHCl_3$ peak in $CDCl_3$ at 77.23 or to the residual CD_3OH peak in CD_3OD at 49.5 ppm. The following abbreviations are used for peak multiplicities: app (apparent), s (singlet); br s (broadened singlet); d (doublet); dd (doublet of doublets); ddd

(doublet of doublet of doublets); dt (doublet of triplets); tt (triplet of triplets) t (triplet); q (quartet); quin (quintet); m (multiplet). Coupling constants, *J*, are reported in Hertz (Hz). IR spectra were recorded on a Brucker FT-IR spectrometer. High-resolution mass spectra (HRMS) were obtained on an AccuTOF instrument equipped with a DART ionization source. Melting points were observed in open Pyrex capillary tubes and are uncorrected. Specific rotations $[\alpha]_D$ were obtained on a JASCO polarimeter using the sodium D-line as a source, and the concentration (c) is expressed in g per 100 mL.

Flash chromatography was performed on Silica Gel, 40 micron, 32-63 flash silica from Sorbent. Thin layer chromatography was performed on silica gel (Silicycle Silica Gel 60 F_{254} glass plates). Compounds were visualized by UV, 5% phosphomolybdic acid in ethanol, 0.5% potassium permanganate in water or a solution of ethanol/H_2SO_4/AcOH/*p*-anisaldehyde (135:5:1.5:3.7). Ceric molybdate in a solution of H_2O/ammonium molybdate/ceric ammonium molybdate/ H_2SO_4 (235 mL: 12 g: 0.5 g: 15mL) was used for sulfatides.

Preparation of 15*Z*,18*Z*-Tetracosadienoic acid (VI)

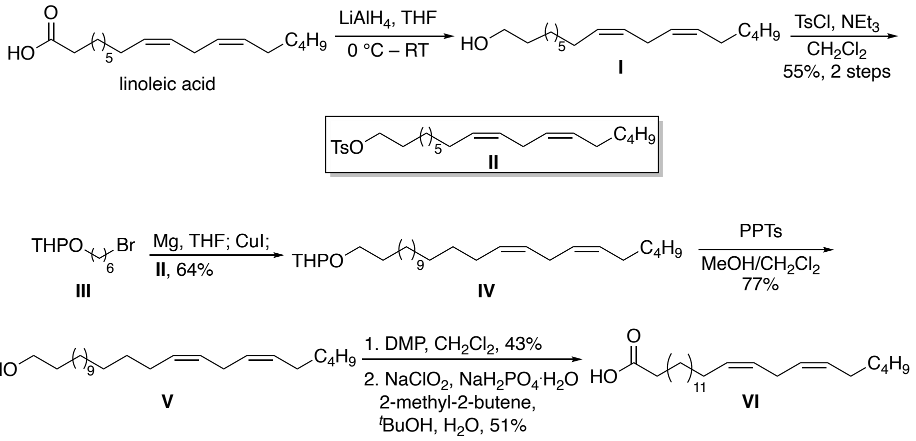

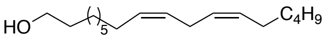

I

9*Z*,12*Z*-Octadecadien-1-ol (I). Linoleic acid (1.0 g, 3.5 mmol) was dissolved in dry THF (40 mL) under N_2, and the solution was cooled to 0 °C. After 10 min, $LiAlH_4$ (2M in THF, 5.3 mL, 10.6 mmol) was added dropwise over 2 min. The solution was stirred at 0 °C for 1 h then was allowed to warm to rt over 2.5 h. The reaction mixture was then cooled to 0 °C, and the excess $LiAlH_4$ was carefully quenched with saturated aqueous NH_4Cl (20 mL). The organic layer was separated, and the aqueous layer was extracted with EtOAc (3 x 30 mL). The combined organic extracts were dried ($MgSO_4$) and concentrated. The crude colorless oil (**I**) was moved forward without purification. ^{1}H NMR (400 MHz, $CDCl_3$) δ 5.41−5.30 (m, 4H), 3.64−3.60 (m, 2H), 2.78 (t, J = 6.3 Hz, 2H), 2.13–2.10 (brs, 1H), 2.08−2.03 (m, 4H), 1.59−1.53 (m, 2H), 1.40−1.29 (m, 16 H), 0.90 (t, J = 6.0 Hz, 3H); ^{13}C NMR (100 MHz, $CDCl_3$) δ 130.2, 130.1, 128.0, 127.9, 62.8, 32.8, 31.5, 29.7, 29.5, 29.4, 29.3, 29.2, 27.2, 27.2, 25.8, 25.6, 22.6, 14.0.

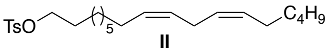

II

9*Z*,12*Z*-Octadecadiene tosylate (II). Triethyl amine (0.60 mL, 4.3 mmol) and DMAP (48 mg, 0.40 mmol) were added to 9Z,12Z-octadecadien-1-ol (**I**) (1.0 g, 3.9 mmol) in dry CH_2Cl_2 (3.8 mL) at 0 °C. After 10 min, TsCl (0.78 g, 4.1 mmol) was added, and the solution was allowed to warm to rt overnight. The reaction was diluted with CH_2Cl_2 (15 mL) and washed with saturated aqueous NH_4Cl (25 mL). The organic layer was separated, and the aqueous layer was extracted with CH_2Cl_2 (3 x 25 mL). The combined organic extracts were dried ($MgSO_4$) and concentrated. Purification via flash column chromatography (hexanes/EtOAc 95:5) yielded **II** as a colorless oil (0.92 g, 55% over two steps): IR (neat) 2927, 2857, 1357, 1174 cm^{-1}; ^{1}H NMR (400 MHz, $CDCl_3$) δ 7.80 (d, J = 8.2 Hz, 2H), 7.35 (d, J = 8.1 Hz, 2H), 5.43−5.31 (m, 4H), 4.04 (t, J = 6.5

Hz, 2H), 2.79 (t, *J* = 6.2 Hz, 2H), 2.46 (s, 3H), 2.09–2.03 (m, 4 H), 1.68-1.61 (m, 2H), 1.41−1.23 (m, 16H), 0.89 (t, *J* = 6.7 Hz, 3H); ^{13}C NMR (100 MHz, $CDCl_3$) δ 144.6, 133.3, 130.2, 130.0, 129.8, 128.1, 127.9, 127.8, 70.6, 31.5, 29.6, 29.4, 29.3, 29.1, 28.9, 28.8, 27.2, 27.2, 25.6, 25.3, 22.6, 21.6, 14.1.

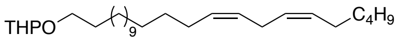

IV

1-(2-Tetrahydro-2H-pyranyl)oxy-15*Z*,18*Z*-tetracosadiene (IV). Magnesium turnings (0.11 g, 5.0 mmol) were added to a flame dried 3-neck round bottom equipped with a reflux condenser. The flask was flame dried a second time before adding a crystal of I_2 and dry THF (4.5 mL). 2-[(6-Bromohexyl)oxy]tetrahydro-2H-pyran (**III**)(1) (1.2 g, 4.6 mmol), was added in two portions. Approximately one third of III was added to the flask, which was then heated with a heat gun until the solution turned colorless. Once the color disappeared, remaining III was added while maintaining reflux with the heat gun. The round bottom was then placed in a 60 °C oil bath to stir for 40 min. The Grignard reagent was then added dropwise over 5 min to a suspension of CuI (0.43 g, 2.3 mmol) in dry THF (7.5 mL) at 0 °C. The reaction mixture was stirred at 0 °C for 40 min. 9*Z*,12*Z*-Octadecadiene tosylate (II) (0.48 g, 1.1 mmol) in dry THF (7.5 mL) was added dropwise over 5 min, and the mixture was stirred for 5 h at 0 °C, then allowed to warm to rt. Saturated aqueous NH_4Cl (25 mL) was added, and the aqueous layer was extracted with Et_2O (3 x 30 mL). The combined organic extracts were washed with brine (40 mL), dried ($MgSO_4$) and concentrated. Purification via flash column chromatography (Hexanes/EtOAc 99:1) on silica gel yielded **IV** as a pale yellow oil (0.35 g, 64%): IR (neat) 3009, 2922, 2852, 1033 cm^{-1}; ^{1}H NMR (400 MHz, $CDCl_3$) δ 5.42–5.31 (m, 4H), 4.60–4.58 (m, 1H), 3.88 (ddd, *J* = 10.9, *J* = 7.4, *J* = 2.8 Hz, 1H), 3.75 (ddd, *J* = 9.6, *J* = 6.9, *J* = 6.9 Hz, 1H), 3.54–3.48 (m, 1H), 3.40 (ddd, *J* = 9.5, *J* =

6.6, J = 6.6 Hz, 1H), 2.79 (t, J = 6.4 Hz, 2H), 2.09–2.04 (m, 4H), 1.89−1.81 (m, 1H), 1.76−1.70 (m, 1H), 1.64−1.50 (m, 6H), 1.41−1.29 (m, 28H), 0.89 (t, J = 6.5 Hz, 3H); ^{13}C NMR (100 MHz, $CDCl_3$) δ 130.1, 128.0, 127.9, 98.8, 67.7, 62.2, 31.5, 30.8, 29.8, 29.7, 29.6, 29.6, 29.5, 29.4, 29.3, 27.2, 27.2, 26.3, 25.6, 25.5, 22.6, 19.7, 14.0; HRMS (ESI) calcd for $C_{29}H_{55}O_2$ $[M + H]^+$ *m/z* 435.4202, found 435.4169.

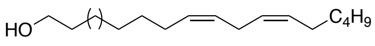

V

15*Z*,18*Z*-Tetracosadien-1-ol (V). 1-(2-Tetrahydro-2H-pyranyl)oxy-15*Z*,18*Z*-tetracosadiene (**IV**) (0.35 g, 0.81 mmol) was dissolved in CH_2Cl_2/MeOH (1:1, 3.8 mL) followed by the addition of PPTS (20 mg, 0.081 mmol). The reaction was stirred at 45 °C for 8 h. The MeOH was evaporated, and the residue was diluted with CH_2Cl_2 (20 mL) and H_2O (20 mL). The organic layer was separated, and the aqueous layer was extracted with CH_2Cl_2 (3 x 10 mL). The combined organic extracts were dried ($MgSO_4$) and concentrated. Purification via flash column chromatography (hexanes/EtOAc 95:5) yielded **V** as a pale yellow oil (0.22 g, 77%): IR (neat) 3400 (br), 2917, 2849, 1462, 1071, 683 cm^{-1}; ^{1}H NMR (400 MHz, $CDCl_3$) δ 5.41−5.30 (m, 4H), 3.64 (t, J = 6.6 Hz, 2H), 2.77 (t, J = 6.5 Hz, 2H), 1.57 (quin, J = 7.3 Hz, 4H), 1.39−1.26 (m, 31H), 0.89 (t, J = 6.6 Hz, 3H); ^{13}C NMR (100 MHz, $CDCl_3$) δ 130.4, 128.2, 33.0, 31.8, 29.8, 29.8, 29.8, 29.7, 29.6, , 29.6, 27.5, 27.4, 26.0, 25.9, 14.3; HRMS (ESI) calcd for $C_{24}H_{47}O$ $[M + H]^+$ *m/z* 351.3621, found 351.3621.

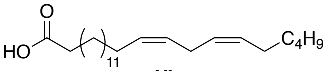

VI

15*Z*,18*Z*-Tetracosadienoic acid (VI). Dess-Martin periodinane (0.16 g, 0.38 mmol) was added to a solution of 15*Z*,18*Z*-tetracosedien-1-ol (**V**) (0.12 g, 0.35 mmol) in dry CH_2Cl_2 (1.3 mL) at 0 °C. The reaction mixture was stirred at rt for 6h. The reaction mixture was filtered through a pad of celite, and the celite was washed with CH_2Cl_2 (10 mL). The combined filtrates were concentrated and purified by flash column chromatography on silica gel (hexanes/EtOAc, 90:10) to provide 15*Z*,18*Z*-tetracosadienal as a colorless oil (53 mg, 43%): IR (neat) 2920, 2850, 1700, 1650, 1510, 1100, 1050 cm^{-1}; ^{1}H NMR (400 MHz, $CDCl_3$) δ 9.76 (s, 1H), 5.41–5.30 (m, 4H), 2.77 (t, J = 6.0 Hz, 2H), 2.41 (t, J = 7.2 Hz, 2H), 2.04 (m, 4H), 1.61 (m, 2H), 1.34–1.26 (m, 26H), 0.89 (t, J = 6.9 Hz, 3H); ^{13}C NMR (100 MHz, $CDCl_3$) δ 203.2, 130.4, 128.2, 44.1, 31.8, 29.9, 29.9, 29.8, 29.6, 29.6, 29.6, 29.4, 27.5, 27.4, 25.9, 22.8, 22.3, 14.3. NaH_2PO_4 (0.14 g, 1.0 mmol) was added to a mixture of 15*Z*,18*Z*-tetracosadienal (0.060 g, 0.18 mmol) and 2-methyl-2-butene (0.4 mL, 4 mmol) in ^{t}BuOH (7 mL) and H_2O (1.5 mL) at 0 °C. $NaClO_2$ (0.020 g, 0.22 mmol) was added in small portions and the mixture stirred for 6 h. One more equiv of $NaClO_2$ was added, and the reaction mixture was left in the fridge overnight. The next day, TLC still showed remaining aldehyde; so another equiv of $NaClO_2$ was added, and the reaction mixture was stirred for 40 min at 0 °C. After this, TLC showed complete consumption of the aldehyde. Saturated aqueous Na_2SO_3 and pH7 phosphate buffer (1:1, 2 mL) were added. The product was extracted with EtOAc (3 X 10 mL). The combined organic extracts were washed with sat. NH_4Cl (5 mL) and brine (5 mL), dried ($MgSO_4$), filtered and concentrated to give **VI** with ~10% of inseparable *E/Z*-stereoisomers (0.036 g, 51%) as a colorless oil: ^{1}H NMR (400 MHz, $CDCl_3$) δ 5.41–5.30 (m, 4H), 2.77 (t, J = 5.8 Hz, 2H), 2.34 (t, J = 7.5 Hz, 2H), 2.05 (m, 4H), 1.63 (quin, J = 7.2 Hz, 2H), 1.40–1.26 (m, 26H), 0.91–0.86 (m, 3H); ^{13}C NMR (100 MHz, $CDCl_3$) δ 180.5,

130.4, 128.2, 34.3, 31.8, 30.0, 29.9, 29.8, 29.7, 29.6, 29.6, 29.5, 29.3, 27.5, 27.4, 25.8, 24.9, 22.8, 14.3.

General *p*-nitrophenyl ester preparation

p-Nitrophenol (1.1 equiv) and DMAP (0.2 equiv.) were added to a flask charged with carboxylic acid (1.0 equiv.) in dry CH_2Cl_2 (0.014 M), and the solution was stirred for 15 min. DCC (1.04 equiv) in dry CH_2Cl_2 (0.12 M) was then added slowly. The reaction mixture was allowed to stir at rt overnight, then filtered through a pad of celite. The celite was washed with CH_2Cl_2, and the filtrate was concentrated. Purification via flash chromatography on silica gel (petroleum ether/EtOAc, 95:5) yielded PNP-activated esters **VII-IX**.

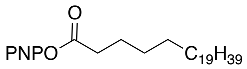

VII

***p*-Nitrophenyltetracosanoate (VII).** Compound **VII** was prepared from tetracosanoic acid and was isolated as a white solid (0.29 g, 73%): mp 81.9–82.2 °C; IR (neat) 2916, 2849, 1752, 1535, 1347, 1203, 1136, 1107, 927, 868, 717 cm^{-1}; ^{1}H NMR (400 MHz, $CDCl_3$) δ 8.29–8.25 (m, 2H), 7.29–7.26 (m, 2H), 2.59 (t, J = 7.4 Hz, 2H), 1.76 (quin, J = 7.3 Hz 2H), 1.45–1.26 (m, 40H), 0.88 (t, J = 7.0 Hz, 3H); ^{13}C NMR (100 MHz, $CDCl_3$) δ 171.5, 155.8, 145.4, 125.4, 122.6, 34.6, 32.2, 29.9, 29.8, 29.7, 29.6, 29.4, 29.3, 25.0, 22.9, 14.3; HRMS (ESI) calcd for $C_{30}H_{52}NO_4$ $[M + H]^+$ *m/z* 490.3891, found 490.3921.

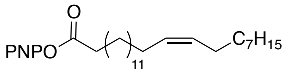

VIII

***p*-Nitrophenyl 15*Z*-tetracosenoate (VIII).** Compound **VIII** was prepared from nervonic acid and was isolated as a colorless solid (0.50 g, 73%): mp 35.5–36.0 °C; IR (neat) 2916, 2850, 1753, 1593, 1536, 1490, 1471, 1350, 1203, 1138, 926, 868, 717 cm^{-1}; ^{1}H NMR (400 MHz, $CDCl_3$) δ 8.18 (d, *J* = 8.7 Hz, 2H), 7.19 (d, *J* = 8.6 Hz, 2H), 5.27 (m, 2H), 2.51 (t, *J* = 7.3 Hz, 2H), 1.96–1.91 (m, 4H), 1.68 (quin, *J* = 7.0 Hz, 2H), 1.34–1.19 (m, 32H), 0.80 (t, *J* = 6.7 Hz, 3H); ^{13}C NMR (100 MHz, $CDCl_3$) δ 171.4, 155.7, 145.4, 130.1, 130.0, 125.3, 122.6, 34.5, 32.1, 30.0, 29.8, 29.8, 29.7, 29.6, 29.5, 29.4, 29.2, 27.4, 24.9, 22.9, 14.3; HRMS (ESI) calcd for $C_{30}H_{50}NO_4$ $[M + H]^+$ *m/z* 488.3734, found 488.3755.

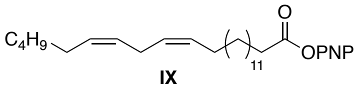

IX

***p*-Nitrophenyl 15*Z*,18*Z*-tetracosedienoate (IX).** Compound **IX** was prepared from 15*Z*,18*Z*-tetracosadienoic acid (**VI**) and was isolated as a low melting solid (29.0 mg, 45%): IR (neat) 2922, 2852, 1768, 1593, 1524, 1490, 1464, 1345, 1208, 1098, 863 cm^{-1}; ^{1}H NMR (400 MHz, $CDCl_3$) δ 8.18 (d, *J* = 9.0 Hz, 2H), 7.19 (d, *J* = 8.9 Hz, 2H), 5.33–5.21 (m, 4H), 2.71–2.64 (m, 2H), 2.52 (t, *J* = 7.4 Hz, 2H), 1.97 (m, 4H), 1.68 (quin, *J* = 7.2 Hz, 2H), 1.36–1.20 (m, 26H), 0.81 (t, *J* = 6.1 Hz, 3H); ^{13}C NMR (100 MHz, $CDCl_3$) δ 171.4, 155.7, 145.4, 130.3, 128.1, 125.3, 122.6, 34.5, 31.7, 29.8, 29.8, 29.6, 29.5, 29.4, 29.2, 27.6, 27.4, 25.8, 24.9, 22.8, 14.2; HRMS (ESI) calcd for $C_{30}H_{48}NO_4$ $[M + H]^+$ *m/z* 486.3578, found 486.3570.

Preparation of sulfatides C24:0 and C24:2

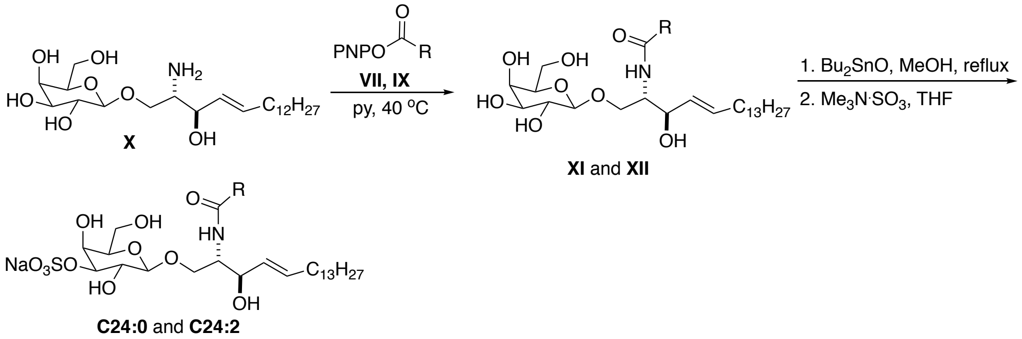

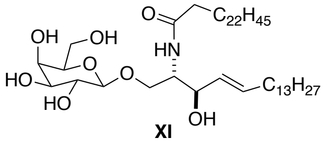

XI

(2*S*,3*R*,4*E*)-1-(β-D-Galactopyranosyloxy)-2-(*N*-tetracosanoylamino)octadec-4-en-3-ol (XI). *p*-Nitrophenyltetracosaneoate (**VII**) (0.04 g, 0.08 mmol) was added to a solution of (2*S*,3*R*,4*E*)-2-amino-1-(β-galactopyranosyloxy)octadec-4-en-3-ol(2) (**X**) (0.03 g, 0.7 mmol) in pyridine (1 mL). The solution was stirred in a preheated oil bath at 40 °C overnight. The solution was concentrated and purified by flash column chromatography on silica gel (CH_2Cl_2/MeOH, 90:10) to give **XI** as a white solid (17.2 mg, 32%): mp 182.0–183.0 °C; $[\alpha]^{25}_D$ 1.10 (c 1.25, $CHCl_3$/MeOH); ^{1}H NMR (400 MHz, $CDCl_3$/CD_3OD, 3:2) δ 7.27 (d, *J* = 8.8 Hz, 1H), 5.69 (dt, *J* = 15.2, 7.2 Hz, 1H), 5.45 (dd, *J* = 15.0, 6.6 Hz, 1H), 4.21 (d, *J* = 6.8 Hz, 1H), 4.00 (br s, 1H), 3.88 (br s, 1H), 3.81–3.72 (m, 2H), 3.61 (m, 1H), 3.56–3.50 (m, 3H), 2.77 (t, *J* = 6.9 Hz, 2H), 2.02 (dt, *J* = 6.9, 6.9 Hz, 2H), 1.60–1.57 (m, 2H), 1.36–1.26 (m, 62H), 0.88 (t, *J* = 6.4 Hz, 6H); ^{13}C NMR (100 MHz, $CDCl_3$/CD_3OD, 3:2) δ 175.5, 135.0, 130.2, 104.7, 76.0, 74.4, 73.0, 72.3, 70.0, 69.6, 62.5, 54.5, 50.1, 33.2, 32.7, 30.5, 30.5, 30.4, 30.3, 30.2, 30.2, 26.8, 23.5, 14,6; HRMS (ESI) calcd for $C_{48}H_{94}NO_8$ $[M + H]^+$ *m/z* 812.6974, found 812.6982.

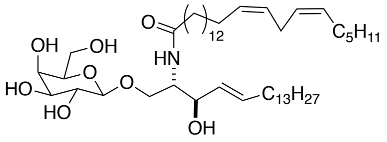

XII

(2*S*,3*R*,4*E*)-1-(β-D-Galactopyranosyloxy)-2-(*N*-15*Z*,18*Z*-tetracosadienoylamino)octadec-4-en-3-ol (XII). *p*-Nitrophenyl 15*Z*,18*Z*-tetracosadieneoate (**IX**) (28 mg, 0.06 mmol) was added to a solution of (2*S*,3*R*,4*E*)-2-amino-1-(β-galactopyranosyloxy)octadec-4-en-3-ol(2) **(X)** (25 mg, 0.60 mmol) in pyridine (1 mL). The mixture was stirred in a preheated oil bath at 40 °C overnight. The reaction was concentrated and purified by flash column chromatography on silica gel (CH_2Cl_2/MeOH, 90:10) to give **XII** (21 mg, 46%) as an off white solid: mp 129.0–130.0 °C; IR (neat) 3302, 2915, 1641, 1544, 1467, 1082 cm^{-1}; ^1H NMR (400 MHz, $CDCl_3/CD_3OD$, 3:2) δ 5.70 (dt, *J* = 14.6, 6.6 Hz, 1H), 5.46 (m, 1H), 5.41–5.29 (m, 4H), 4.21 (d, *J* = 7.4 Hz, 1H), 4.00 (ddd, *J* = 7.3, 3.7, 3.7 Hz, 1H), 3.82 (app d, *J* = 2.6 Hz, 1H), 3.81 (dd, *J* = 11.5, 6.6 Hz, 1H), 3.75 (dd, *J* = 11.5, 5.0 Hz 1H), 3.62 (dd, *J* = 10.3, 3.2 Hz, 1H), 3.57–3.47 (m, 3H), 2.77 (t, *J* = 6.2 Hz, 2H), 2.17 (t, *J* = 7.4 Hz, 2H), 2.07–1.99 (m, 6H), 1.59 (quin, *J* = 7.1 Hz, 2H), 1.40–1.27 (m, 53H), 0.88 (t, *J* = 6.9 Hz, 6H); ^{13}C NMR (100 MHz, $CDCl_3/CD_3OD$, 3:2) δ 175.6, 135.0, 131.0, 130.3, 128.9, 104.8, 76.1, 74.5, 73.1, 72.4, 70.0, 69.7, 62.5, 54.5, 37.4, 33.2, 32.8, 32.4, 30.5, 30.5, 30.4, 30.3, 30.3, 30.2, 28.1, 28.1, 26.8, 26.5, 23.5, 23.4, 14.7; HRMS (ESI) calcd for $C_{48}H_{90}NO_8$ $[M + H]^+$ *m/z* 808.6661, found 808.6660.

General sulfation procedure

Glycolipids (1 equiv) and Bu_2SnO (1.2 equiv) were refluxed in MeOH (0.016 M) for 2 h. The solvent was evaporated under reduced pressure. The resulting dibutyl-stannylene complex was treated with $Me_3N{\bullet}SO_3$ (2 equiv) in THF (2 mL)(3, 4). The mixture was stirred at rt from

between 2 and 6 h. TLC was used to monitor the reaction. The solvent was evaporated, and the residue dissolved in a 1:1 mixture of CH_3Cl_3/MeOH (4 mL). Dowex (Na^+ resin) was added. The mixture was then stirred for 10 min, followed by filtration and concentration. The crude product was partitioned in a mixture of 1-butanol/H_2O (1:1, v/v) and centrifuged. The supernatant (1-butanol, containing the sulfatides) was collected and concentrated. Purification by flash column chromatography on silica gel (CH_2Cl_2/MeOH, 90:10 – 85:15) gave the sulfatides.

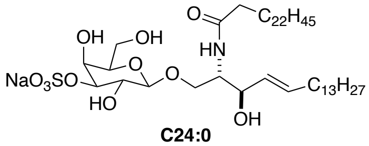

C24:0

(*2S,3R,4E*)-1-(3-*O*-Sodiumsulfonyl-β-D-galactopyranosyloxy)-2-(*N*-tetracosanoylamino)-octadec-4-en-3-ol (C24:0). Following the general sulfation procedure, sulfatide **C24:0** was isolated as a white solid (4.2 mg, 35%): mp 204.0–205.0 ºC; $[\alpha]^{25}_{D}$ 6.98 (*c* 0.38, $CHCl_3$/MeOH, 3:2); IR (neat) 3400 (br), 2917, 2850, 1646, 1466, 1258, 1066 cm^{-1}; ^{1}H NMR (400 MHz, $CDCl_3$/CD_3OD, 3:2) δ 7.71 (d, J = 8.8 Hz, 1H), 5.67 (dt, J = 15.3, 6.7 Hz, 1H), 5.42 (dd, J = 15.3, 6.6 Hz, 1H), 3.81–3.74 (m, 4H), 3.58–3.56 (m, 2H), 2.15 (t, J = 7.9 Hz, 2H), 2.00–1.98 (m, 2H), 1.66–1.45 (m, 2H), 1.41–1.25 (m, 62H), 0.87 (t, J = 6.9 Hz, 6H); ^{13}C NMR (100 MHz, $CHCl_3$/CD_3OD, 3:2) δ 175.7, 135.4, 130.3, 104.3, 81.4, 75.8, 72.7, 70.5, 69.7, 68.2, 62.2, 54.2, 37.4, 33.4, 32.9, 31.5, 30.7, 30.6, 30.6, 30.5, 30.4, 30.3, 30.3, 26.9, 23.5, 14.8; HRMS (TOF) *m/z* calcd for $C_{48}H_{92}NO_{11}S$ [M –Na]$^+$ 890.6397, found 890.6377.

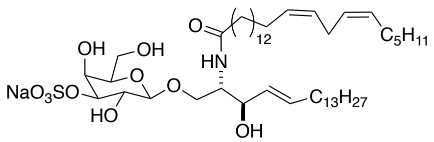

C24:2

(2*S*,3*R*,4*E*)-1-(3-*O*-Sodiumsulfonyl-β-D-galactopyranosyloxy)-2-(*N*-15*Z*,18*Z*-tetracosadienoylamino)octadec-4-en-3-ol (C24:2). Sulfatide **C24:2** was isolated as an off white solid (14.9 mg, 65%): mp 182.0–183.0 °C; IR (neat) 3370 (br), 2918, 2850, 1644, 1467, 1258, 1066 cm^{-1}; ^{1}H NMR (400 MHz, $CDCl_3/CD_3OD$, 3:2) δ 5.70 (dt, *J* = 15.3, 6.6 Hz, 1H), 5.44 (dd, *J* = 15.4, 7.4 Hz, 1H), 5.40–5.29 (m, 4H), 4.34 (d, *J* = 7.7 Hz, 1H), 3.64 (dd, *J* = 10.3, 3.2 Hz, 1H), 3.57 (dd, *J* = 5.7, 5.7 Hz, 1H), 2.77 (d, *J* = 6.3 Hz, 2H), 2.17 (t, *J* = 7.6 Hz, 2H), 2.08–2.00 (m, 6H), 1.65–1.51 (m, 2H), 1.40–1.27 (m, 48H), 0.91–0.86 (m, 6H); ^{13}C NMR (100 MHz, $CHCl_3/CD_3OD$, 3:2) δ 175.8, 135.2, 131.1, 130.4, 128.9, 104.5, 81.4, 75.8, 72.9, 70.6, 69.9, 68.6, 62.4, 54.5, 37.4, 33.2, 32.8, 32.4, 30.6, 30.5, 30.5, 30.4, 30.4, 30.3, 30.2, 28.1, 28.1, 26.9, 26.6, 23.5, 23.4, 14.7; HRMS (TOF) calcd for $C_{48}H_{92}NO_{11}$ [M –Na]$^{+}$ *m/z* 886.6078, found 886.6058.

Preparation of pC24:0, pC24:1 and pC24.2

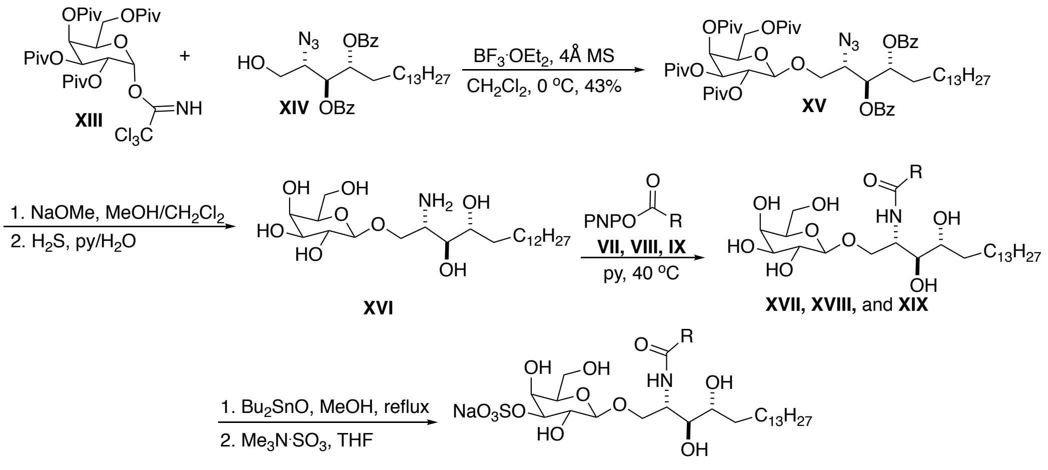

pC24:0, **pC24:1** and **pC24:2**

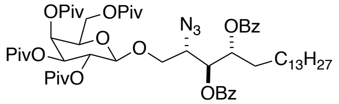

XV

(2*S*,3*S*,4*R*)-2-Azido-3,4-dibenzoyloxy-1-(2,3,4,6-tetra-*O*-pivaloyl-β-D-galactopyranoside)octadecane (XV). (2,3,4,6-Tetra-*O*-pivaloyl-α-D-galactopyranoside)-1-trichloroacetimidate(2) (**XIII**) (0.60 g, 0.99 mmol) and (2*S*,3*S*,4*R*)-2-azido-(3,4-dibenzoyloxy)octadecan-1-ol(5) (**XIV**) (0.45 g, 0.82 mmol) were dissolved in dry CH_2Cl_2 (13 mL), and the solution was stirred in the presence of 4Å MS (600 mg) at rt for 10 min. The solution was then cooled to –10 ºC. BF_3•OEt_2 in dry CH_2Cl_2 (1.46 μL in 2 mL) was added over 10 min; then the solution was allowed to slowly warm to rt and stir for 1.5 h. The reaction mixture was diluted with petroleum ether (50 mL) and then filtered. The filtrate was washed with saturated aqueous $NaHCO_3$ (10 mL). The organic layer was separated, and the aqueous phase was extracted with CH_2Cl_2 (3 X 15 mL). The combined organic extracts were dried ($MgSO_4$), filtered and concentrated. Purification by flash column chromatography on silica gel (petroleum ether/ EtOAc 95:5) gave **XV** (0.34 g, 39%) as a colorless oil: $[\alpha]^{25}_{D}$ –3.65 (*c* 1.00, CH_2Cl_2); IR (neat) 2926, 2103, 1728, 1480, 1261, 1140, 710 cm^{-1}; ^{1}H NMR (500 MHz, $CDCl_3$) δ 8.00 (d, *J* = 7.8 Hz, 4H), 7.57 (m, 2H), 7.44 (m, 4H), 5.49– 5.44 (m, 2H), 5.37 (d, *J* = 3.1 Hz, 1H), 5.22 (dd, *J* = 10.5, 8.1 Hz, 1H), 5.05 (dd, *J* = 10.4, 3.2 Hz, 1H), 4.56 (d, *J* = 7.9 Hz, 1H), 4.08–4.02 (m, 2H), 3.98–3.90 (m, 4H), 1.88–1.80 (m, 2H), 1.43–1.32 (m, 3H), 1.29–1.20 (m, 30H), 1.14 (s, 9H), 1.09 (s, 9H), 1.08 (s, 9H), 0.86 (t, *J* = 6.7 Hz, 3H). ^{13}C NMR (125 MHz, $CDC1_3$) δ 177.9, 177.4, 177.0, 176.5, 133.7, 133.4, 130.0, 129.9, 129.8, 129.5, 128.7, 128.6, 100.9, 73.0, 71.2, 71.1, 68.7, 68.6, 66.7, 61.4, 61.1, 39.2, 38.9, 38.8, 32.0, 30.3, 29.8, 29.8, 29.8, 29.8, 29.8, 29.7, 29.7, 29.6, 29.5, 29.5, 29.5, 25.4, 22.8, 14.2; HRMS (ESI) calcd for $C_{58}H_{88}N_3O_{14}$ $[M + H]^+$ *m/z* 1050.6261, found 1050.6300.

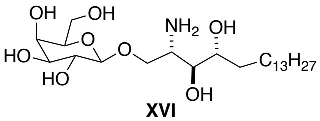

XVI

(2*S*,3*S*,4*R*)-2-Amino-1-(β- D-galactopyranosyloxy)octadecan-3,4-diol (XVI). NaOMe in MeOH (0.50 M, 4.0 mL, 2.0 mmol) was added to a solution of (2*S*,3*S*,4*R*)-2-azido-3,4-dibenzoyloxy-1-(2,3,4,6-tetra-*O*-pivaloyl-β-galactopyranoside)octadecane (**XV**) (296 mg, 0.28 mmol) in a mixture of CH_2Cl_2/MeOH (3.4/3.4 mL)(2). The solution was stirred at rt for 1.5 h. The solution was then acidified with dowex (H^+) resin. The mixture was filtered through a pad of celite, and the celite was washed with a 1:1 mixture of $CHCl_3$ and MeOH (15 mL). The filtrate was concentrated and triturated with petroleum ether/EtOAc (85:15) to give (2*S*,3*S*,4*R*)-2-azido-1-(β-galactopyranosyloxy)octadecan-3,4-diol (134 mg, 94%) as a white solid: $[\alpha]^{25}_D$ 18.9 (*c* 6.64, $CHCl_3$/MeOH, 3:2); IR (neat) 3355 (br), 2915, 2849, 2096, 1255, 1071 cm^{-1}; ^{1}H NMR (400 MHz, $CDCl_3/CD_3OD$, 3:2) δ 4.28 (d, *J* = 7.2 Hz, 1H), 4.13 (dd, *J* = 10.6, 5.0 Hz, 1H), 3.96 (app d, *J* = 10.3 Hz, 1H), 3.97 (s, 1H), 3.82 (dd, *J* = 11.5, 6.5 Hz, 1H), 3.70–3.63 (m, 4H), 3.58–3.49 (m, 3H), 1.67–1.56 (m, 2H), 1.42–1.25 (m, 24H), 0.87 (t, *J* = 6.8 Hz, 3H). ^{13}C NMR (100 MHz, $CDC1_3/CD_3OD$, 3:2) δ 104.3, 76.1, 74.9, 74.4, 72.7, 72.1, 69.9, 69.4, 63.1, 62.3, 33.2, 32.8, 30.6, 30.5, 30.5, 30.2, 26.6, 23.5, 14.7; HRMS (ESI) calcd for $C_{24}H_{48}N_3O_8$ $[M + H]^+$ *m/z* 506.3436, found 506.3511. The product was carried forward to reduction of the azide. A solution of (2*S*,3*S*,4*R*)-2-azido-1-(β-galactopyranosyloxy)octadecan-3,4-diol (13 mg, 0.27 mmol) in a mixture of pyridine/H_2O (1:1, 7.6 mL) was saturated with H_2S. The solution was stirred for 48 h(2). The solvent was evaporated to give **XVI** (136 mg, crude) as a yellowish brown powder, which was carried forward without purification.

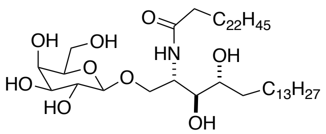

XVII

(2*S*,3*S*,4*R*)-1-(β-D-Galactopyranosyloxy)-2-(*N*-tetracosanoylamino)octadecane-3,4-diol (XVII). *p*-Nitrophenyltetracosanoate (**VII**) (30 mg, 0.07 mmol) was added to a solution of (2*S*,3*S*,4*R*)-2-amino-1-(β-galactopyranosyloxy)octadecane-3,4-diol (**XVI**) (30 mg, 0.06 mmol) in pyridine (1 mL). The mixture was stirred in a preheated oil bath at 40 ^{o}C overnight. The reaction was concentrated and purified by flash column chromatography on silica gel (CH_2Cl_2/MeOH, 95:5) to give **XVII** as a white solid (16 mg, 35%): mp 198.7–199.8 ^{o}C; $[\alpha]^{25}_{D}$ 10.20 (*c* 0.49, $CHCl_3$/MeOH, 3:2); IR (neat) 3304, 2915, 2849, 1625, 1468, 1077, 718 cm^{-1}; ^{1}H NMR (400 MHz, $CDCl_3$/MeOD, 3:2) δ 4.25–4.12 (m, 2H), 3.87–3,86 (m, 1H), 3.82 (dd, *J* = 11.6, 6.7 Hz, 2H), 3.75–3.69 (m, 2H), 3.61–3.47 (m, 5H), 2.20 (t, *J* = 7.5 Hz, 2H), 1.68–1.52 (m, 4H), 1.44–1.27 (m, 64H), 0.88 (t, *J* = 6.5 Hz, 6H); ^{13}C NMR (100 MHz, $CDCl_3$/MeOD, 3:2) δ 175.6, 104.8, 76.3, 75.4, 74.5, 73.3, 72.4, 70.2, 70.1, 51.5, 37.4, 33.2, 32.8, 31.3, 30.7, 30.6, 30.6, 30.5, 30.3, 30.3, 30.2, 26.8, 26.8, 23.5, 14.7; HRMS (TOF) *m/z* calcd for $C_{48}H_{96}NO_9$ $[M + H]^+$ 830.7080, found 830.7052.

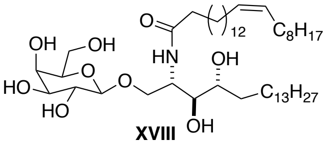

XVIII

(2*S*,3*S*,4*R*)-1-(β-D-Galactopyranosyloxy)-2-(*N*-15*Z*-tetracosenoylamino)octadecan-3,4-diol (XVIII). *p*-Nitrophenyl 15*Z*-tetracosenoate (**VIII**) (33 mg, 0.07 mmol) was added to a solution of (2*S*,3*S*,4*R*)-2-amino-1-(β-galactopyranosyloxy)octadecan-3,4-diol (**XVI**) (30 mg, 0.06 mmol) in pyridine (1 mL). The mixture was stirred in a preheated oil bath at 40 ^{o}C overnight. The

reaction was concentrated and purified by flash column chromatography on silica gel (CH_2Cl_2/MeOH, 95:5) to give **XVIII** (19 mg, 38%) as a white solid: mp 169.0–171.0 °C; $[\alpha]^{25}_D$ 7.38 (*c* 0.82, $CHCl_3$/MeOH, 3:2); IR (neat) 3330, 2917, 2849, 1637, 1545, 1465, 1081, 1049, cm^{-1}; ^{1}H NMR (400 MHz, $CDCl_3$/CD_3OD, 3:2) δ 5.38–5.30 (m, 2H), 4.24–4.20 (m, 2H), 4.16 (dd, *J* = 10.3, 4.6 Hz, 1H), 3.87 (app d, *J* = 2.7 Hz, 1H), 3.82 (dd, *J* = 11.6, 6.8 Hz, 1H), 3.74 (ddd, *J* = 11.1, 4.8 Hz, 1H), 3.71 (dd, J = 10.2, 3.8 Hz, 1H), 3.61–3.47 (m, 5H), 2.20 (t, *J* = 7.6 Hz, 2H), 2.04–1.99 (m, 4H), 1.68–1.57 (m, 3H), 1.53–1.51 (m, 1H), 1.47–1.27 (m, 58H), 0.88 (t, *J* = 6.0 Hz, 6H); ^{13}C NMR (100 MHz, $CDCl_3$/CD_3OD, 3:2) δ 175.5, 130.7, 104.8, 76.2, 75.5, 74.5, 73.3, 72.4, 70.1, 70.1, 62.6, 51.5, 37.4, 33.6, 32.7, 30.6, 30.6, 30.5, 30.4, 30.3, 30.3, 30.2, 30.1, 28.0, 26.8, 26.7, 23.5, 14.6; HRMS (TOF) *m/z* calcd for $C_{48}H_{94}NO_9$ $[M - H]^+$ 828.6923, found 828.6915.

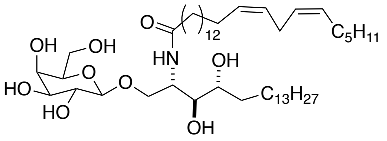

XIX

(2*S*,3*S*,4*E*)-1-(β- D-Galactopyranosyloxy)-2-(*N*-15*Z*,18*Z*-tetracosadienoylamino)octadecan-3.4-diol (XIX). *p*-Nitrophenyl 15*Z*,18*Z*-tetracosadieneoate (**IX**) (28 mg, 0.06 mmol) was added to a solution of (2*S*,3*S*,4*R*)-2-amino-1-(β-galactopyranosyloxy)octadecan-3,4-diol (**XVI**) (27.0 mg, 0.06 mmol) in pyridine (1 mL). The mixture was stirred in a preheated oil bath at 40 °C overnight. The reaction was concentrated and purified by flash column chromatography on silica gel (CH_2Cl_2/MeOH, 95:5) to give **XIX** (19 mg, 40%) as a white solid: mp 169.0–171.0 °C; $[\alpha]^{25}_D$ 8.81 (c 1.86, $CHCl_3$/MeOH, 3:2); IR (neat) 3302, 2918, 2850, 1637, 1467, 1082 cm^{-1}; ^{1}H NMR (400 MHz, $CDCl_3$/CD_3OD, 3:2) δ 5.41–5.29(m, 4H), 4.23–4.22 (m, 2H), 4.14–4.12 (m, 1H),

3.87 (app d, *J* = 2.0 Hz, 1H), 3.82 (dd, *J* = 11.6, 6.8 Hz, 1H), 3.75–3.69 (m, 2H), 3.61–3.47 (m, 5H), 2.77 (t, *J* = 6.1 Hz, 2H), 2.20 (t, *J* = 7.4 Hz, 2H), 2.07–2.01 (m, 4H), 1.68–1.51 (m, 5H), 1.44–1.27 (m, 51H), 0.88 (t, *J* = 4.8 Hz, 6H); ^{13}C NMR (100 MHz, $CDCl_3$ $CDCl_3/CD_3OD$, 3:2) δ 175.6, 131.0, 128.9, 76.3, 75.5, 74.5, 73.3, 72.4, 70.1, 70.1, 62.6, 51.5, 37.4, 33.2, 32.8, 32.4, 30.7, 30.6, 30.4, 30.3, 30.3, 30.2, 28.1, 28.1, 26.8, 26.8, 26.5, 23.5, 23.4, 14.7; HRMS (TOF) *m/z* calcd for $C_{48}H_{92}NO_9$ $[M - H]^+$ 826.6767, found 826.6777.

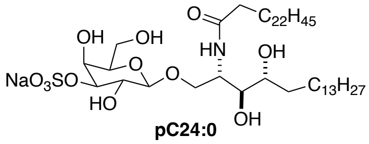

pC24:0

(*2S,3S,4R*)-1-(3-*O*-sodiumsulfonyl-β- D-galactopyranosyloxy)-2-(*N*-tetracosanoylamino)-octadecane-3,4-diol (pC24:0). The general sulfation procedure was followed, and sulfatide **pC24:0** was isolated as a white solid (3.2 mg, 18%): mp 184.0.0–185.0 °C; $[\alpha]^{25}_{D}$ 10.43 (*c* 0.49, $CHCl_3$/MeOH, 3:2); IR (neat) 3429 (br), 2917, 2850, 1632, 1467, 1224, 1070, 801 cm^{-1}; ^{1}H NMR (400 MHz, $CDCl_3/CD_3OD$, 3:2) δ 5.35–5.32 (m, 1H), 3.81 (dd, *J* = 11.8, 7.2 Hz, 1H), 3.76–3.71 (m, 2H), 3.66–3.64 (m, 1H), 3.59–3.56 (m, 1H), 2.15 (t, *J* = 7.5 Hz, 2H), 2.04–2.00 (m, 1H), 1.60–1.51 (m, 4H), 1.41–1.26 (m, 62H), 0.87 (t, *J* = 7.0 Hz, 6H); ^{13}C NMR (100 MHz, $CDCl_3/CD_3OD$) δ 175.8, 104.2, 81.2, 75.8, 74.8, 73.2, 70.5, 70.1, 68.3, 62.3, 51.2, 37.3, 32.8, 32.6, 30.6, 30.6, 30.4, 30.3, 30.2, 28.0, 26.9, 26.8, 23.5, 14.8; HRMS (TOF) *m/z* calcd for $C_{48}H_{94}NO_{12}S$ $[M - Na]^+$ 908.6502, found 908.6465.

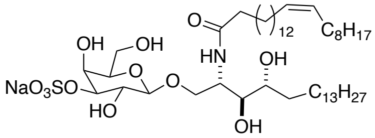

pC24:1

(*2S,3S,4R*)-1-(3-*O*-Sodiumsulfonyl-β- D-galactopyranosyloxy)-2-(*N*-15*Z*-tetracosenoyl-amino)octadecane-3,4-diol (pC24:1). The general sulfation procedure was followed, and sulfatide **pC24:1** was isolated as a white solid (5.3 mg, 58%): mp 211.4–212.4 ºC; $[\alpha]^{25}_{D}$ 8.33 (*c* 0.50, $CHCl_3$/MeOH, 3:2); IR (neat) 3367 (br), 2917, 2850, 1643, 1466, 1224, 1066, 812 cm^{-1}; ^{1}H NMR (400 MHz, $CDCl_3/CD_3OD$, 3:2) δ 7.78 (m, 1H), 5.37–5.30 (m, 2H), 4.34 (d, *J* = 7.7 Hz, 1H), 4.29-4.24 (m, 2H) 3.81 (dd, *J* = 11.*8*, 3.1 Hz, 1H), 3.76–3.56 (m, 2H), 3.70–3.64 (m, 2H), 3.59–3.57 (m, 2H), 2.20 (t, *J* = 7.6 Hz, 2H), 2.04–2.00 (m, 4H), 1.64–1.50 (m, 4H), 1.44–1.26 (m, 56H), 0.87 (t, *J* = 6.4 Hz, 6H); ^{13}C NMR (100 MHz, $CDCl_3/CD_3OD$ 3:2) δ 175.8, 130.8, 104.2, 81.3, 75.8, 74.9, 73.2, 70.5, 70.1, 68.3, 62.3, 51.2, 37.4, 37.3, 32.9, 32.8, 30.7, 30.7, 30.6, 30.6, 30.5, 30.4, 30.4, 30.3, 30.2, 30.2, 28.1, 27.0, 26.9, 23.6, 14.8; HRMS (TOF) *m/z* calcd for $C_{48}H_{90}NO_{12}S$ $[M - Na]^+$ 906.6340, found 906.6339.

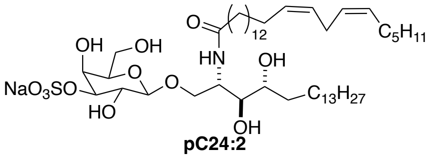

pC24:2

(2*S*,3*S*,4*R*)-1-(3-*O*-Sodiumsulfonyl-β- D-galactopyranosyloxy)-2-(*N*-15*Z*,18*Z*-tetraco-sadienoylamino)octadecan-3,4-diol (pC24:2). The general sulfation procedure was followed, and sulfatide **pC24:2** (containing small amounts of alternative acyl chain isomers) was isolated as an off white solid (8.0 mg, 48%): mp 172.0−173.0 ºC; IR (neat) 3400 (br), 2917, 2850, 1637, 1467, 1226, 1061 cm^{-1}; ^{1}H NMR (500 MHz, $CDCl_3/CD_3OD$, 3:2) δ 5.41–5.30 (m, 4H), 3.81–3.78 (m, 1H), 3.75–3.72 (m, 2H), 3.69–3.64 (m, 2H), 3.60–3.57 (m, 2H), 2.77 (t, *J* = 6.6 Hz,

1H), 2.20 (t, *J* = 7.3 Hz, 2H), 2.07–2.02 (m, 4H), 1.59–1.52 (m, 4H), 1.38–1.26 (m, 54H), 0.88 (m, 6H); ^{13}C NMR (125 MHz, $CDCl_3/CD_3OD$, 3:2) δ 175.8, 131.1, 131.0, 128.9, 128.9, 104.2, 81.3, 75.8, 74.8, 73.3, 70.5, 70.1, 68.3, 62.3, 54.4, 37.3, 37.3, 32.8, 32.6, 32.4, 30.6, 30.5, 30.4, 30.3, 30.2, 28.1, 28.1, 26.9, 26.8, 26.5, 23.5, 23.4, 14.8; HRMS (TOF) calcd for $C_{48}H_{92}NO_{12}S^-$ $[M – Na]^+$ *m/z* 904.6189, found 904.6210.

Preparation of SR-21-177B and SR-22-24A

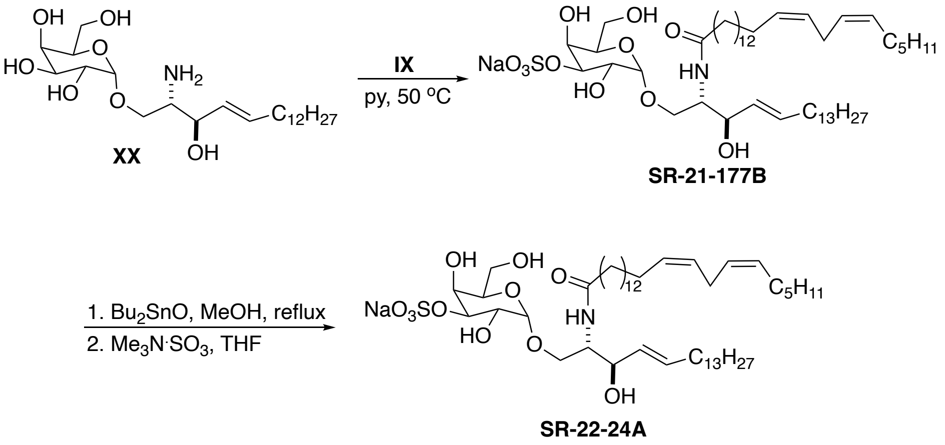

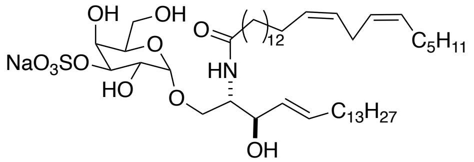

SR-21-177B

(2*S*,3*S*,4*E*)-1-(α-D-Galactopyranosyloxy)-2-(*N*-15*Z*,18*Z*-tetracosadienoylamino)octadecan-3.4-diol (SR-21-177B). *p*-Nitrophenyl 15*Z*,18*Z*-tetracosadieneoate (**IX**) (36 mg, 0.074 mmol) was added to a solution of (2*S*,3*S*,4*R*)-2-amino-1-(α-galactopyranosyloxy)octadecan-3,4-diol (**XX**)(6) (31 mg, 0.067 mmol) in pyridine (2 mL). The mixture was stirred in a preheated oil bath at 50 °C for 24 h. The reaction mixture was concentrated and purified by gravity column chromatography on silica gel (CH_2Cl_2/MeOH, 90:10) to give **SR-21-177B** (26 mg, 48%) as a

white solid: ^{1}H NMR (400 MHz, $CDCl_3/CD_3OD$, 1:1) δ 7.47 (d, *J* = 8.7 Hz, 1H), 5.70 (ddd, *J* = 14.8, 6.7, 6.7 Hz, 1H), 5.38 (dd, *J* = 15.4, 7.0 Hz, 1H), 5.39–5.28 (m, 4H), 4.86, (d *J* = 3.6 Hz, 1H), 4.05 (dd, *J* = 6.9, 6.9 Hz, 1H), 3.96–3.91 (m, 2H), 3.80–3.72 (m, 6H), 2.76 (t, *J* = 6.4 Hz, 2H), 2.18 (t, *J* = 7.6 Hz, 2H), 2.06–1.99 (m, 6H), 1.58–1.56 (m, 2H), 1.36–1.25 (m, 46H), 0.89–0.88 (m, 6H); ^{13}C NMR (100 MHz, $CDCl_3/CD_3OD$, 1:1) 174.5, 133.8, 129.7, 129.0, 127.6, 99.7, 71.8, 70.5, 70.0, 69.5, 68.8, 67.2, 61.4, 53.5, 36.1, 32.1, 31.6, 31.2, 29.4, 29.3, 29.3, 29.2, 29.1, 28.0, 26.9, 26.8, 25.7, 25.3, 22.3, 22.2 13.5, 13.5; HRMS (ESI) calcd for $C_{48}H_{90}NO_8$ $[M + H]^+$ *m/z* 808.6667, found 808.6691.

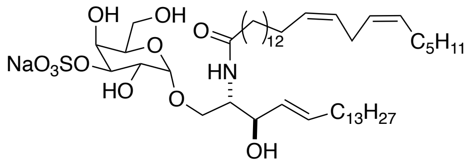

SR-22-24A

(2*S*,3*R*,4*E*)-1-(3-*O*-Sodiumsulfonyl-α-D-galactopyranosyloxy)-2-(*N*-15*Z*,18*Z*- tetracosa-dienoylamino)octadec-4-en-3-ol (SR-22-24A). The general sulfation procedure was followed, and sulfatide **SR-22-24A** was isolated as a white solid (13 mg, 45%): ^{1}H NMR (400 MHz, $CDCl_3/CD_3OD$, 1:1) δ 7.67 (d, *J* = 8.9 Hz, 1H), 5.73 (ddd, *J* = 14.8, 7.2, 7.2 Hz, 1H), 5.44 (dd, *J* = 15.4, 7.2 Hz, 1H), 5.39–5.28 (m, 4H), 4.92, (d *J* = 3.8 Hz, 1H), 4.49 (dd, *J* = 10.2, 3.1 Hz, 1H), 4.34 (m, 1H), 4.11 (dd, *J* = 7.5, 7.5 Hz, 1H), 4.00 (dd, *J* = 10.3, 3.8 Hz, 1H), 3.97–3.93 (m, 1H), 3.84 (dd, J = 5.6, 5.6 Hz, 1H), 3.80–3.72 (m, 4H), 2.76 (t, *J* = 6.3 Hz, 2H), 2.20 (t, *J* = 7.6 Hz, 2H), 2.07–2.00 (m, 6H), 1.58–1.56 (m, 2H), 1.35–1.25 (m, 46H), 0.89–0.86 (m, 6H); ^{13}C NMR (100 MHz, $CDCl_3/CD_3OD$, 1:1) δ 174.9, 134.1, 129.9, 129.9, 129.4, 127.8, 99.6, 77.771.5, 70.6, 68.2, 67.3, 66.9, 61.6, 53.7, 36.3, 32.3, 31.8, 31.4, 29.6, 29.5, 29.4, 29.4, 29.3, 29.2, 29.2, 27.1,

27.1, 25.9, 25.4, 22.5, 22.4, 13.7, 13.6; HRMS (ESI) calcd for $C_{48}H_{88}NNa_2O_{11}S$ $[M + H]^+$ *m/z* 932.5874, found 932.5877.

Reagents

Fluorescent protein labeled monoclonal antibodies used for flow cytometry were obtained as follows: Mouse: anti-CD8α (clone 53-6.7), anti-CD86 (clone GL-1), anti-CD80 (clone 16-10A1), anti-CD70 (clone FR70), anti-PDL1 (clone 10F.9G2), anti-PDL2 (clone TY25), anti-CD45 (clone 30-F11) and anti-CD69 (clone H1.2F3) antibodies were purchased from Biolegend (San Diego, CA, USA). Anti-B220 (clone RA3-6B2), anti-CD3 (clone 145-2C11), anti-CD1d (clone 1B1), anti-CD11c (clone HL3), anti-CD45 (clone 30-F11), anti-TCRβ (clone H57-597), anti-CD11b (clone M1/70) and anti-CD40 (clone 3/23) antibodies were purchased from BD BioSciences (San Jose, CA, USA). Anti CD11c (clone REA754) was purchased from Miltenyi Biotec (Gaithersburg, MD, USA). Anti-CD11b was purchased from eBioscience (San Diego, CA, USA). PBS57 (α-GalCer analogue)-loaded CD1d tetramer was obtained from the NIH Tetramer Core Facility (Emory University, Atlanta, GA, USA). Human: anti-CD3 (clone SP43-2), anti-IFNγ (clone B27) were purchased from BD BioSciences (San Jose, CA, USA), and LIVE/DEAD Fixable Blue Dead Cell Stain was purchased from Invitrogen (Carlsbad, CA, USA).

Supplementary Fig. Legend

Supplementary Fig. S1. The injection of C24:2 stimulated much more cytokine production in serum than C24:1.

Heat map representing color-coded expression levels of cytokine profiles of mice injected i.p. with the vehicle used to dissolve the sulfatide analogues, 500 pmol of KRN7000, or 30 nmol of sulfatide analogues is shown. Serum samples were collected 3 h, 6 h, 12 h, and 24 h after lipid injection and analyzed. n=5 mice per group. Each row represents an individual mouse.

Supplementary Fig. S2. Effects of C24:2 treatment on CD1d surface expression.

BALB/c mice were injected with 30 nmol of C24:2 i.p. CD1d expression of splenocytes was assessed by flow cytometry. Results are representative data from two experiments (mean ± SD) (n=3 mice per group). (**A**) Representative flow plot schematic. (**B**) Quantified MFI of CD1d expression of various $CD45^+$ cells.

Supplementary Fig. S3. Alpha anomer of C24:2 and C24:1.

(**A**) Structures of the alpha-anomer of C24:2 (SR-22-24A), the alpha anomer of bGalCer C24:2 (SR-21-177B), and the alpha-anomer of C24:1 (αC24:1). (**B**) 50,000 BMDC were incubated with glycolipid for 3 h and subsequently co-incubated with DN32 cells at a 1:1 ratio overnight. IL-2 secretion in supernatant from DN32 cells was assessed by ELISA. Results are representative data from two experiments (mean ± SD). (**C**) CD1d-lipid complexes were adhered to 96-well plates and co-cultured with DN32 cells overnight. IL-2 secretion in supernatant from

DN32 cells was assessed via ELISA. Results are representative data from two experiments (mean ± SD).

Supplementary Fig. S4. Representative flow schematic of ICS of human PBMCs. Representative flow schematic of 1x10e6 healthy human PBMCs were cultured with 10 ug/mL of glycolipid (C24:2 with and without BAF 50 nM) for 15 h then 1 h with brefeldin A. Additionally, human PBMCs were cultured with cell activation cocktail in the presence of BAF.

Supplementary References

1. Morimoto Y, Kitao S, Okita T, Shoji T. Total Synthesis and Assignment of the Double-Bond Position and Absolute Configuration of (−)-Pyrinodemin A. Organic Letters. 2003;5(15):2611-4.
2. Zimmermann P, Bommer R, Bare T, Schmidt RR. Glycosylimidates. Part 33. Azidosphinogosine glycosylation in glycosphingolipid synthesis. J Carbohydr Chem. 1988;7(2):435-52.
3. Guilbert B, Davis NJ, Pearce M, Aplin RT, Flitsch SL. Tetrahedron Asymmetry. 1994;5:2163.
4. Compostella F, Franchini L, De Libero G, Palmisano G, Ronchetti F, Panza L. Tetrahedron 2002;58:8703.
5. Veerapen N, Brigl M, Garg S, Cerundolo V, Cox LR, Brenner MB, et al. Synthesis and biological activity of α-galactosyl ceramide KRN7000 and galactosyl (α1→2) galactosyl ceramide. Bioorg Med Chem Lett. 2009;19(15):4288-91.
6. Deng S, Kain L, Pereira CS, Mata S, Macedo MF, Bendelac A, et al. Psychosine variants as antigens for natural killer T cells. Chemical Science. 2017;8(3):2204-8.

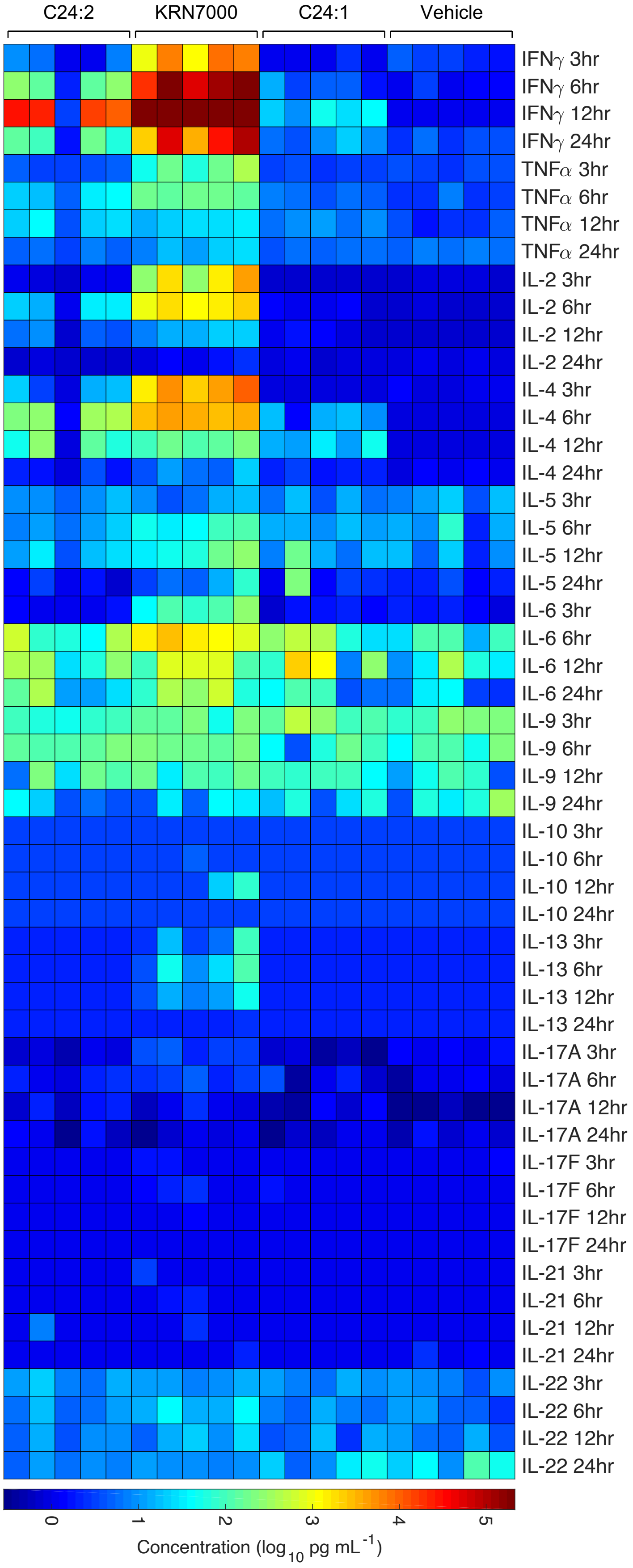

Supplementary Figure S1

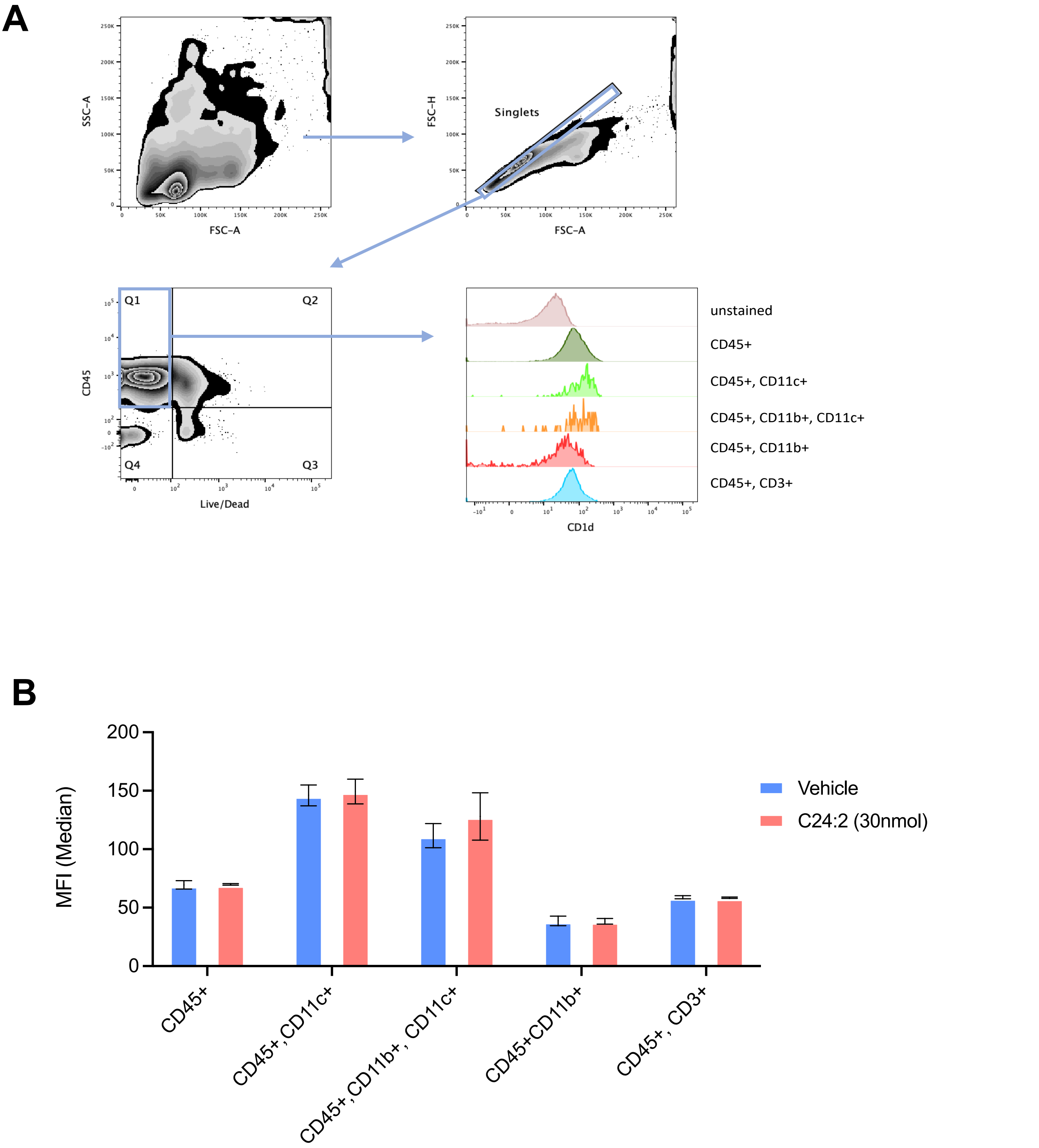

Supplementary Figure S2

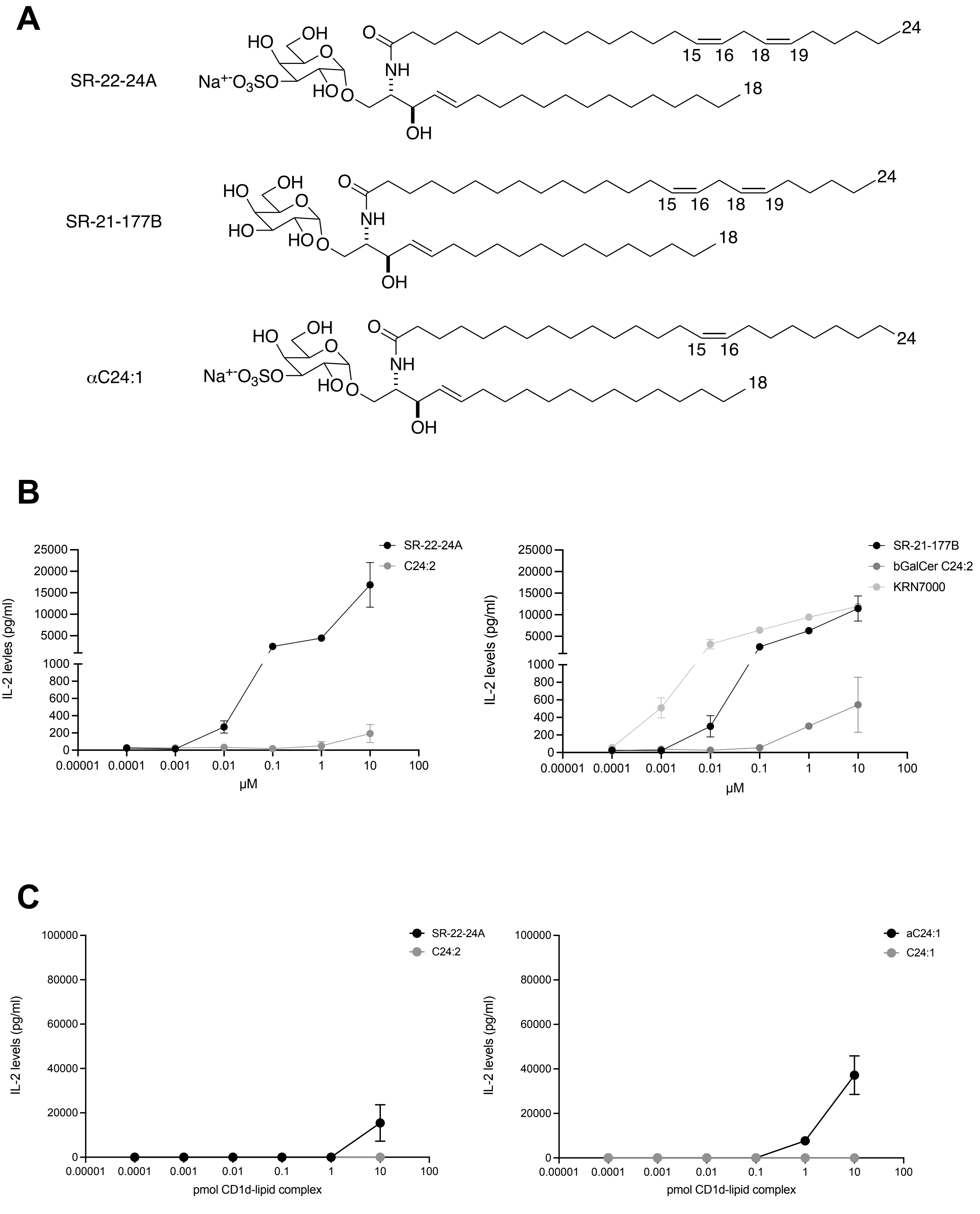

Supplementary Figure S3

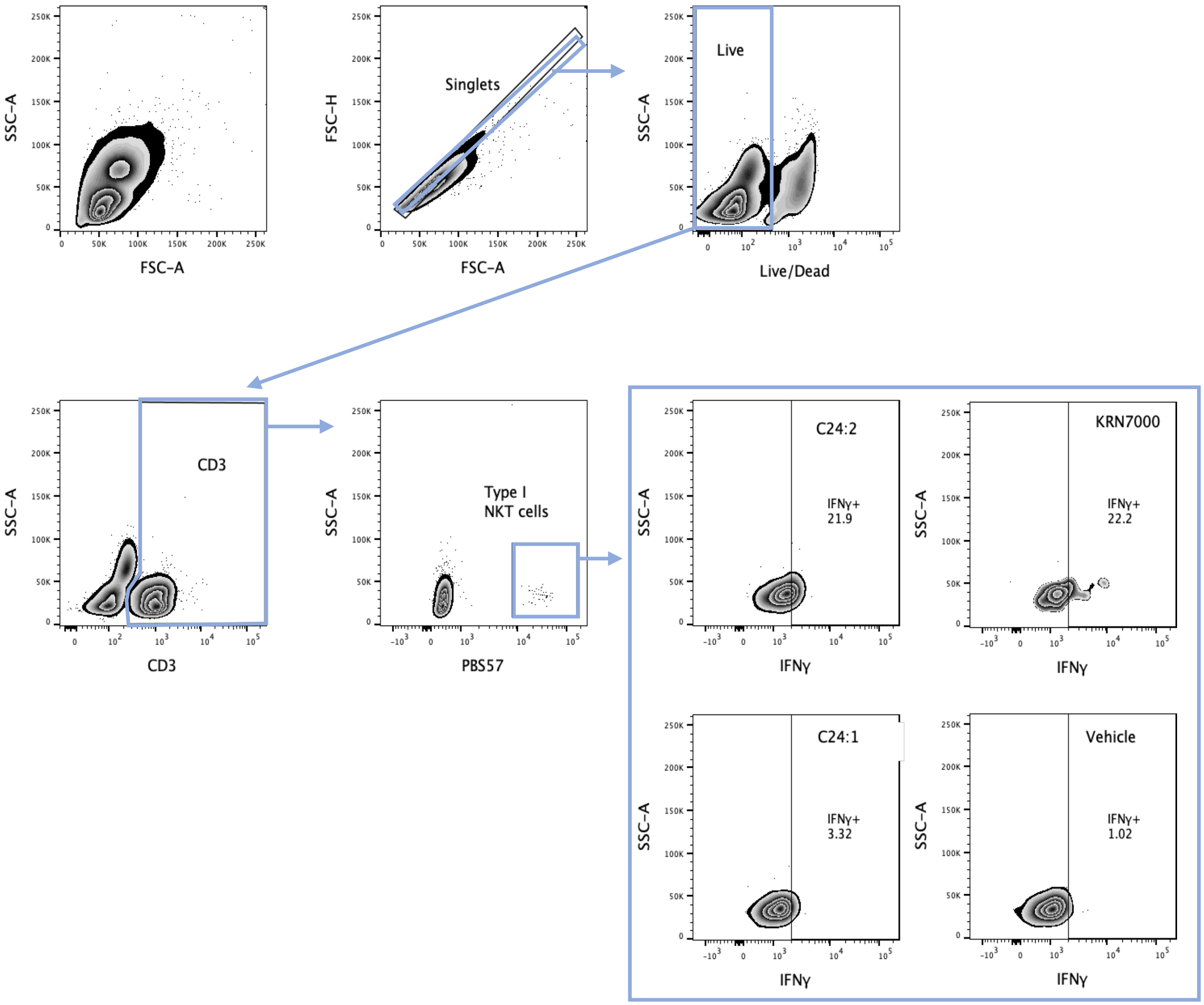

Supplementary Figure S4
